# Multi-robot cooperation for lunar *In-Situ* resource utilization

**DOI:** 10.3389/frobt.2023.1149080

**Published:** 2023-03-23

**Authors:** Bernardo Martinez Rocamora, Cagri Kilic, Christopher Tatsch, Guilherme A. S. Pereira, Jason N. Gross

**Affiliations:** ^1^ Field and Aerial Robotics Laboratory, Department of Mechanical and Aerospace Engineering, Benjamin M. Statler College of Engineering and Mineral Resources, West Virginia University, Morgantown, WV, United States; ^2^ Navigation Laboratory, Department of Mechanical and Aerospace Engineering, Benjamin M. Statler College of Engineering and Mineral Resources, West Virginia University, Morgantown, WV, United States; ^3^ Interactive Robotics Laboratory, Department of Mechanical and Aerospace Engineering, Benjamin M. Statler College of Engineering and Mineral Resources, West Virginia University, Morgantown, WV, United States

**Keywords:** multi-robot systems, aerospace robotics, planetary rovers, Moon, autonomous lunar rover operations

## Abstract

This paper presents a cooperative, multi-robot solution for searching, excavating, and transporting mineral resources on the Moon. Our work was developed in the context of the Space Robotics Challenge Phase 2 (SRCP2), which was part of the NASA Centennial Challenges and was motivated by the current NASA Artemis program, a flagship initiative that intends to establish a long-term human presence on the Moon. In the SRCP2 a group of simulated mobile robots was tasked with reporting volatile locations within a realistic lunar simulation environment, and excavating and transporting these resources to target locations in such an environment. In this paper, we describe our solution to the SRCP2 competition that includes our strategies for rover mobility hazard estimation (e.g. slippage level, stuck status), immobility recovery, rover-to-rover, and rover-to-infrastructure docking, rover coordination and cooperation, and cooperative task planning and autonomy. Our solution was able to successfully complete all tasks required by the challenge, granting our team sixth place among all participants of the challenge. Our results demonstrate the potential of using autonomous robots for autonomous *in-situ* resource utilization (ISRU) on the Moon. Our results also highlight the effectiveness of realistic simulation environments for testing and validating robot autonomy and coordination algorithms. The successful completion of the SRCP2 challenge using our solution demonstrates the potential of cooperative, multi-robot systems for resource utilization on the Moon.

## 1 Introduction

The 16 November 2022, launch of the Artemis 1 mission was a significant milestone in the plans of the United States to return humankind to the surface of the Moon. The long-term human presence on the Moon, as envisioned by the Artemis program (NASA, 2020b; Smith et al., 2020), will require autonomous robotics technologies that support *in-situ* resource utilization (ISRU) in extraterrestrial environments (Colaprete et al., 2017). For example, extracting resources from the lunar soil, such as oxygen and water, will be vital to sustaining humans and building outposts for future missions.

As part of the initiative, NASA is planning a series of progressive robotic missions to the lunar surface. For example, the Volatile Exploring Polar Exploration Rover (VIPER) (Colaprete et al., 2019) is scheduled to land on the Moon’s south pole in 2024 and will explore permanently shadowed regions and examine subsurface material for the presence of volatile content using a drill. This mission will try to confirm the presence of the materials identified in orbital missions. Next, as part of the Artemis program (NASA, 2020b), future rovers and landers will test technologies such as site preparation, robotic mining, and energy storage systems (NASA Space Technology Mission Directorate (STMD), 2020), leading to the establishment of an Artemis Base Camp on the Lunar south pole (Smith et al., 2020). Moreover, NASA’s Cooperative Autonomous Distributed Robotic Explorers (CADRE) mission plans to explore the Lunar surface in 2024 by utilizing cooperative exploration techniques with a group of rovers (Fong, 2021). Cooperative multi-agent missions allow for exploring the regions of interest faster and more efficiently by allocating tasks with dedicated agents, improving the overall autonomy performance, and enabling coordination between multiple robots (Polizzi et al., 2022).

A significant challenge in leveraging multi-robot coordination for planetary surface missions is the lack of consideration for multiple robots due to the mission profiles. This may be attributed to the higher uncertainty associated with the previous exploration missions on Mars and Moon. However, multi-robot coordination is an important aspect of planetary missions, as is reflected in the 2020 NASA Technology Taxonomy (NASA, 2020a). For Lunar ISRU, the use of multiple robots can help to perform tasks more efficiently (Thangavelautham and Xu, 2022). Coordination between the robots can ensure that each robot is working on its assigned task and the overall mission can be completed in less time (Burgard et al., 2005; Stachniss and Stachniss, 2009). Also, by providing redundancy and backup systems, the use of multiple robots, instead of a single one, reduces the risk of system failure (Mahdoui et al., 2018). Finally, since ISRU missions can be complex, by using multi-robot coordination, the scalability of the mission can be increased (Portugal and Rocha, 2013; Khamis et al., 2015).

Aligned with the needs for technology development on multi-robot systems for the Artemis mission, from 2019 to 2021, the NASA Centennial Challenges Program sponsored the NASA Space Robotics Challenge Phase 2 (SRCP2) (NASA Centennial Challenges Program (CCP), 2021). This challenge was an open-prize competition designed to engage the public in developing robot localization, coordination, autonomy, and control technologies for a team of robots dedicated to ISRU in a virtual lunar environment. The challenge’s goal was to develop reliable software to advance the surface mining capabilities of fully autonomous robot teams on the Moon. The challenge field is a simulated lunar environment where a heterogeneous team of robots must cooperate to complete a series of tasks involving searching, collecting, and delivering different types of lunar resources referred to as “volatiles.”

The challenge was divided into a Qualification Round and a Competition Round. The Qualification Round consisted of three tasks to be completed independently. The goal of Task 1 was to explore the lunar environment using an autonomous Scout rover capable of detecting and identifying resources scattered across the map. Using a Hauler and an Excavator rover, the goal of Task 2 was to excavate resources from the ground and haul them back to a base station. The goal of Task 3 was to detect and localize an object randomly placed in the environment, detect a marker on the base station and align a Scout to it. More information on the specifications of this round and the solution proposed by our team was presented in (Kilic et al., 2021).

In the Qualification Round, 114 teams competed to develop solutions for these tasks. Twenty-two teams qualified for the Competition Round, in which the tasks were combined into a single mission, adding another complexity layer given the increased number of interactions between different types of robots. From the Qualification Round to the Competition Round, additional complexities, such as robot power constraints, harsh lighting conditions, and randomized topography were included. In order to execute the Competition round mission, a team of rovers with up to six members could be selected from three types of rovers introduced in the Qualification Round (i.e., Scout, Hauler, and Excavator). An overview of Competition Round tasks and constraints is provided in Section 3 of this paper, and the detailed descriptions can be found in the SRCP2 official rule document (NASA Centennial Challenges Program (CCP), 2021).

In this article, our main objective is to share the solutions developed by our team for the Competition Round, which have the potential to be utilized for multi-robot systems for lunar ISRU and that granted us the sixth place in the challenge (NASA’s Marshall Space Flight Center, 2021), being one of the only seven declared winners. Our team at West Virginia University was also one of the two winning teams composed solely of students and faculty members, along with the team from the University of Adelaide (Sachdeva et al., 2022). This paper provides an in-depth explanation of the implementations for specific robots that are planned to be utilized for future lunar ISRU missions, as well as the strategies used to coordinate the autonomous operation of multiple robots. It addresses a number of significant technical challenges relevant to the true challenges described by NASA, provides a summary of other competitors’ solutions in the SRCP2 challenge, and offers a solution for a lunar multi-robot system along with open-source software. We believe that the information in this manuscript may be useful for researchers and engineers interested in developing similar software systems for lunar ISRU or those willing to participate in similar competitions. The provided code can serve as an initial point of execution, making it easier for other researchers to build upon this work. The main contributions of this paper can be summarized as 1) Techniques for rover mobility hazard estimation (e.g., slippage level, stuck status) and immobility recovery, 2) Techniques for rover-to-rover and rover-to-infrastructure docking maneuvers, 3) Strategies for rover coordination and cooperation, and 4) Strategies for task planning and autonomy for multi-robot lunar ISRU.

The rest of this paper is organized as follows: Section 2 provides a literature review. Section 3 describes the competition and its goals and summarizes our approach for solving the challenge. Section 4 describes each robot’s intrinsic algorithms and architecture. The autonomy and cooperation strategies are presented in Section 5. Section 6 compiles the results associated with our solution. Finally, Section 7 contains conclusions and a summary of ideas for future work.

## 2 Literature review

Lunokhod 1, in 1970, was the first robot to travel on the surface of another celestial body. It was deployed on the Moon and remotely operated from Earth. The challenges of space robotics are still big, but today we can see many other rovers (e.g., Lunokhod 2, Sojourner, Spirit, Opportunity, Curiosity, Perseverance, Zhurong), with different levels of technology, scattered on the Moon and Mars (Skasiadek, 2013). Space robotics is very challenging due to environmental constraints, such as surviving launch and landing and operating in near-vacuum, low gravity, extreme radiation, extreme temperatures, extreme lighting, extreme abrasive and slippery, and extremely remote environments; and system constraints such as high complexity, long lifetime, extreme reliability and safety, limited onboard mass, limited onboard energy, limited communication, limited testability, etc. (Putz, 1998). Many of the needed technologies for planetary surface exploration are only beginning to be studied (Weisbin and Rodriguez, 2000) and one of the frontiers for the science of space robotics exploration is the development of cooperative architectures to control a team of robots to execute tasks that require coordination and physical interaction such as site preparation, resource collection, and remote science. as evidenced by (NASA, 2020a) and exemplified by the Cooperative Autonomous Distributed Robotic Explorers (CADRE) project (Fong, 2021), that could enable future autonomous robotic exploration and is currently being developed by NASA.

Chien et al. (2000) compared three coordinated planning methods for cooperating rovers: 1) centralized planning, 2) central goal allocation with distributed planning, and 3) contract net protocol. The task was to visit a set of science goals using three identical rovers. The idea was to divide goals between the rovers to minimize driving. The central planner provides a simple platform for checking and planning interactions. However, using a central planner comes with some disadvantages. For example, when the environment is uncertain, a central planner does not perform well because it has to monitor the activities, transmit large amounts of data and execute replanning according to unaccounted events. Additionally, when a central planner fails, the mission is interrupted and ceases to provide the desired output. The central goal allocation with distributed planning has a planner for each agent in addition to a central planner. The central planner allocates the tasks based on and each planner develops a more detailed and executable plan. The advantages come from the reduced workload derived from parallelism and faster reaction time due to diminished communication delay. The disadvantage comes from the inability to reassign goals to different rovers. The contract net protocol is based on a central auctioneer distributing goals and rovers bidding for goals as they are offered. It shares advantages and disadvantages with the second approach, however, through the bidding process it takes the rover resources into account.

A coordination strategy, called Control Architecture for Multi-robot Planetary Outposts (CAMPOUT), was proposed by Schenker et al. (2000) to control the mobility and manipulation of multiple robots to perform site construction operations. The architecture is described as hybrid reactive/deliberative, where a high-level planner allocates tasks assuming finite resources and goal constraints and a low-level planner prescribes behaviors for reactive control in tight perception-action feedback loops. The approach is highly distributed, and each robot can operate independently based on its programmed perception-action behaviors. The authors defend that this architecture has great advantages for space robotics due to the efficient use of system resources, parallelization of the task execution, and tolerance to the failure of individual components. Further developments of these strategies were shown in (Schenker et al., 2003).

More recently, Nishida and Wakabayashi (2012) provided an architecture for lunar exploration, part of the Japanese Aerospace Exploration Agency’s plans to deploy rovers and construct an outpost on the Moon. The technical issues that are being mitigated include electrical power management, limited communication, low traction with the soil, inaccurate position determination, the necessity for a high level of autonomy, and manipulation. Their operation consists of a lander that is able to communicate with the multiple rovers and also the ground segment on Earth. The lander provides the paths which are followed by the rovers using their sensing and control capabilities.

To mitigate complex inter-agent constraints during exploration like collisions and inter-robot communication, Staudinger et al. (2018) proposed a distributed coordination algorithm using sampling-based motion planners to plan paths considering robot dynamics, and a distributed decision-making algorithm (max-sum) to obtain the solution that maximizes the utility function defined for the problem. Additionally, they proposed technologies for *in-situ* space exploration missions, more specifically learning a stationary spatial process based on measurements of individual agents, using a wireless system for communication and localization, and using swarm strategies for navigation and exploration.

The framework used by our team and presented in this paper is based on a centralized task planner and decentralized controllers. In fact, similar to our work, most of the teams competing on SRCP2 also opted for a central task planner with decentralized capabilities for each rover. However, each team had its own particularities. Sachdeva et al. (2022) opted for an approach with two teams of three rovers (one instance of each type of rover). In their approach, each rover had localization, scene understanding, locomotion, and specialized capabilities (e.g., exploration, volatile detection, digging, dumping, parking) and a central coordinator that allocated tasks for each of the rovers, synchronizing their actions and tracking the activities of both teams, providing duplicity. Brabec (2021) opted for two independent teams of three rovers (one instance of each type of rover), separated by a minimum distance of 6 m. Each of the Scouts was given half of the map to explore using a circular lawn-mower pattern, once they found resources they served as a landmark for their companion Excavator and Hauler.

Team Capricorn (2021) opted for a centralized strategy planner with a team-level finite state machine (FSM), global map manager, global resource recorder, global health manager, time estimator, and scheduler. At the individual robot level, they had additional FSMs, a health monitor, and packages for planning, SLAM, and perception. They also had specialized packages for each type of rover: resource finder, arm, and bin controllers. Team L3 (2022) opted for only using one rover of each type. Their goal was to achieve scoring cycles of 20 min. Decision-making and most of the autonomous behavior (manipulation, volatile search, volatile hauling, and power management) were performed by a central planner. Only local planning and object detection were performed individually for each rover.

While these teams approached the same challenge, there was virtually no overlap in how they realized their mission capabilities when compared to our solution in terms of subsystem design, rover coordination, and mission planning. Therefore, the purpose of this paper is to share the details of our specific subsystem implementations and overall mission strategy.

In summary, the field of space robotics is rapidly advancing, with new solutions and technologies being proposed to overcome the challenges of autonomous mobility, coordination, and control of multiple rovers. Methods to coordinate multi-robots, such as centralized planning, central goal allocation with distributed planning; and architectures for multi-robot space missions will be essential for achieving the goals of the Artemis program and its plans for utilizing autonomous robots on the Moon. The goals of the SRCP2 align with the need for technology development for the Artemis program. To better understand the challenges that the SRCP2 aimed to address, we provided an overview of the challenge and our approach in the following section.

## 3 NASA space robotics challenge phase 2 (SRCP2)

The challenge’s goal was to develop reliable software to advance the surface mining capabilities of a fully autonomous team of robots on the Moon. This section provides a high-level description of the challenge and our approach to solving it.

### 3.1 Overview of the challenge—SRCP2 competition round

**FIGURE 1:**
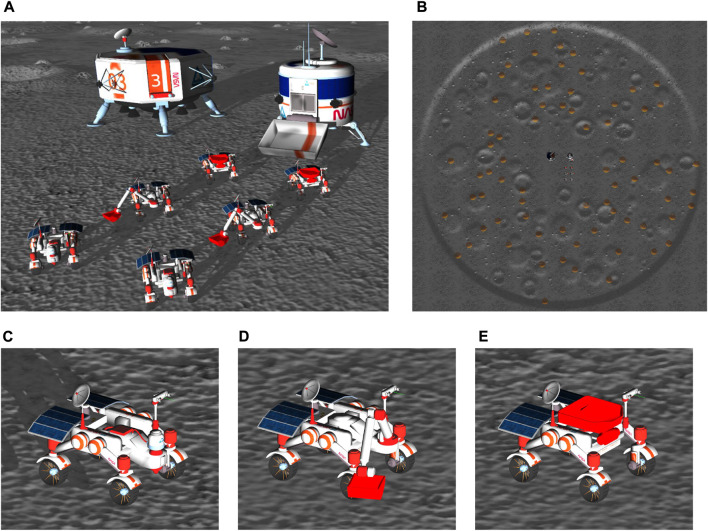
Competition simulation setup. **(A)** Rover initial positions. **(B)** Randomized lunar terrain consisting of one large crater defining the task area, multiple smaller craters, plateaus, obstacles, and volatile resources hidden under the surface. Rover types: **(C)** Scout. **(D)** Excavator. **(E)** Hauler.

The simulation environment for the Competition Round, shown in Figure 1, consisted of a 200 m × 200 m lunar terrain map with randomly generated elevation topography, obstacles, and sparse underground resources (i.e., volatiles). Two landmarks, a processing plant where resources should be deposited, and a charging station, were initialized in a fixed position in the center region of the map. The bearing angles of these two landmarks were sampled from an angle interval, guaranteeing that they were mostly aligned with the illuminated side of the map but stipulating some randomization. To fulfill the mission requirements, a heterogeneous team of up to six rovers could be selected from three types of rovers: Scout, Hauler, and Excavator (see Figures 1C–E). Each rover had unique capabilities to assist in the lunar ISRU mission task. The Scout rover was equipped with a sensor package to detect volatile resources of different types underground. The Excavator rover included a 4 degrees of freedom robotic arm with a bucket end-effector to collect the resources. The Hauler rover included a truck bed to transport the resources to the processing plant.

The rovers were actuated through an interface with simulated controllers using the Robot Operating System (ROS) (Open Robotics, 2022), as specified by the competition rules, and were initialized near the landmarks in a flat portion of the map. Their positions were fixed and their orientations were the same for all the robots but randomized from (0, 2*π*]. All the rovers had similar dimensions (i.e., they fit a 2 m × 2 m×2 m bounding box). Each rover had four wheels that could be controlled by velocity commands. Each wheel had independent steering that could be controlled by joint angle commands, thus allowing different driving modes. The maximum speed of the rovers was about 1.5 m s^−1^. All rovers included a 5 Hz frame rate stereo camera that offered 640 × 480 pixels of resolution and a 2D laser sensor, also at 5 Hz, with 150^◦^ wide field of view and 15 m maximum range mounted on a mast. The mast can rotate by 360^◦^ and tilt by 120^◦^. All rovers included an inertial measurement unit (IMU). All sensors were corrupted with simulated sensor noise. The competitors were prohibited from modifying the rovers’ hardware or simulation parameters.

An additional challenge of the competition was the battery limitation of each rover. Batteries discharged as the rovers drove or actuated their joints (e.g., camera pole and arm joints). If the battery dropped below 30%, the robot was automatically set to a power-saving mode. At this mode, all the sensors were deactivated, and motion commands were set to 10% maximum capability of the robot. Fast charging could be achieved by approaching the charging station. Slow charging could be attained in illuminated regions by aligning the rover solar panels with the direction of the light. Each run of the competition was two hours (2 h) long in simulated real-time. In the simulation, rover communication was simplified such that no communication limitation or latency was modeled among rovers. The information of all rovers was accessible on a centralized system (in a single ROS master), and the only limitations on communication were limits with respect to operations throughput for real-time processing.

Apart from the shared capabilities, each portion of the mission task had its complexity and constraints, in an effort to simulate a realistic lunar ISRU mission in a virtual environment. For instance, to simulate the excavation process, the robotic arm collision with the terrain was disabled. Then, if the Excavator rover’s bucket was within a specific angle range and below a certain depth threshold, five particles, called clods, were spawned inside the bucket. Depending on how close the center of the bucket was to the position of the volatile resources on the map, the competition’s volatile simulator would determine how many of the clods were volatile or regolith. The clods had simulated collision physics and mass. Once in the bucket, the simulated clods could fall to the ground if not appropriately handled. Any clod that fell to the ground was considered lost and disappeared from the simulation. Once excavated from the ground, the volatiles must be placed in the Hauler’s bin to be transported to the processing plant.

A scoring system was defined to reward the retrieval of volatile clods (NASA Centennial Challenges Program (CCP), 2021). Points were scored when resources were delivered to the processing plant by a Hauler. A minimum quantity of a given volatile type was required before it counted as points. The top ten scoring competitors in the Competition Round that meet or exceed the given threshold score (35) would be awarded prizes. Additionally, all the robots had to be within bounds at the end of the simulation to obtain a valid score. Due to the size of the map, limited time, number of resources, and limited robot speed, teams were expected to develop a strategy to enable robot teams to maximize the number of points.

In summary, the main constraints and challenges of the SRCP2 competition included:

• The absence of a GPS or similar satellite-based lunar localization system.
• The need for fully autonomous rovers due to the lack of interactions with a human operator during runtime.
• The constraints of using exteroceptive sensors that simulate the noisy data acquisition in lunar conditions, which are coupled on the mast (e.g., when the camera tilts to some degree, the 2D LiDAR tilts with it).
• The limitations of using a single stereo camera in a low-texture, high-contrast environment with permanently shadowed areas that impair visual odometry (VO) performance.
• The difficulties of navigating in terrain with steep slopes and obstacles, which cause significant slippage and prevent safe rover operation.
• The limitation of detecting volatiles at short-range distances and only using Scout rovers.
• The management of rover battery charge and discharge.
• The difficulties of handling self-collisions and collisions between rovers or with the stations.

### 3.2 Overview of our solution concept

Our overall SRCP2 solution concept utilized a six robots configuration consisting of two of each robot type: Scout, Hauler, and Excavator. Each of the six individual robots had a common set of core capabilities (e.g., localization, navigation, object detection, driving control, and recovery behaviors) developed irrespective of robot type and are described in detail in Section 4. Each type of robot had its actions controlled by a finite state machine (FSM) customized for each of the three robot type’s particular missions. The overall mission execution was controlled by a centralized task planner responsible for assigning target waypoints to the six robots.

Each robot type had a distinct mission that was carried out by leveraging both its core capabilities and its unique mission-specific capabilities. The two Scouts’ mission was to search for resources and create a centralized map of volatiles. Scouts were deployed to opposite sides of the field to maximize search area and to reduce the potential for robot collisions. To maintain accurate localization and charge the battery, each Scout routinely performed homing with respect to the charging station. Each Scout’s waypoint plan was loaded *a priori* and consisted of a simple search heuristic that took into consideration the need to regularly perform homing updates with respect to the base station. Once volatile resources were identified, they were stored in a shared resource map that was continuously updated. Following up, the task planner allocated a team, composed of one Excavator and one Hauler, to collect the resources. The allocation was dependent on the idleness of the excavators and tried to minimize the distance traveled by the excavators to move to the excavation sites. Once arriving at the excavation site, the Excavator would identify a safe parking place for their companion Hauler and then begin excavation. Once an Excavator found volatile material, they commanded their companion Hauler to perform a precise parking maneuver and proceed with excavation once confirming the location of the Hauler. Finally, each Hauler was responsible for traversing to an excavation site, precisely parking next to an Excavator at a dig site, and returning volatiles to the processing plant. After a volatile deposit, the Hauler would charge its batteries in the charging station nearby and perform a homing update to reduce its localization errors. In parallel, Excavators would be moving to the following excavation site, and Scouts continuously searched and mapped volatile locations.

The success of our mission concept heavily relies on the ability to maintain accurate localization and reliable object detection and recognition. These capabilities were crucial for the execution of complex tasks such as approaching recognized objects for parking, dumping, charging, and localization recovery. Object recognition was also used to avoid stationary and moving obstacles, such as rocks and other robots, during navigation toward a goal on the map. To enhance the robustness of our solution, we implemented multiple layers of redundancy through multiple methods to confirm specific conditions, detect failures, and retry procedures using different strategies. These measures were thoroughly tested to ensure their effectiveness. The next two sections describe the technologies developed at the rover level and at the team level.

## 4 Robot systems

**FIGURE 2:**
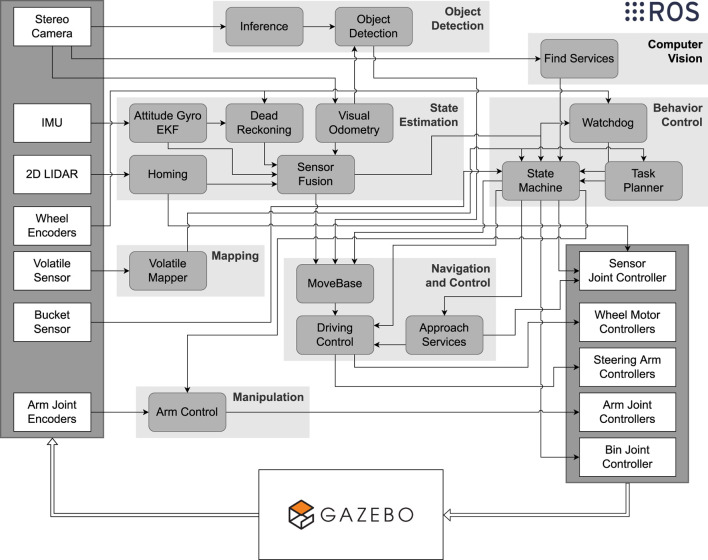
Systems architecture. The virtual environment, simulated using Gazebo, provides access to the sensor data illustrated by the white rectangles on the left side of the figure. The ROS nodes, represented by the rounded gray rectangles, were developed to control the robots and interact with each other to produce setpoints for the controllers made accessible from the simulation.

Many systems and capabilities had to be developed to operate the robots autonomously for two hours: object detection, state estimation, computer vision, behavior control, mapping, manipulation, and navigation and control. The rounded rectangles in Figure 2 show the ROS nodes that compose these systems and their interconnections. In this figure, it is also possible to see how the software interacted with the simulation, i.e., receiving sensor data and outputting setpoints for the controllers. In this section, a brief overview of the approaches developed by our team is provided. These approaches are further discussed in Section 5, where specific usage cases and illustrative examples provide better insights.

### 4.1 Localization and navigation

The challenges of planetary localization and navigation were evident in the provided lunar virtual environments, such as the lack of a continuous global localization source, high slippage on wheels (Asnani et al., 2009), scattered and numerous rocks (Zakrajsek et al., 2005), and varying lighting conditions (Fong et al., 2008). To overcome these challenges, the competition provided a sensor bundle consisting of a stereo camera, 2D LiDAR, IMU, and wheel encoder sensors. In addition to using the sensor bundle for localization and navigation, competitors were given a one-time opportunity to retrieve the rovers’ global position and orientation (pose) information for the rovers in the virtual map during the simulation runtime. This simulated pose fix was reported from a Lunar orbiter.

**FIGURE 3:**
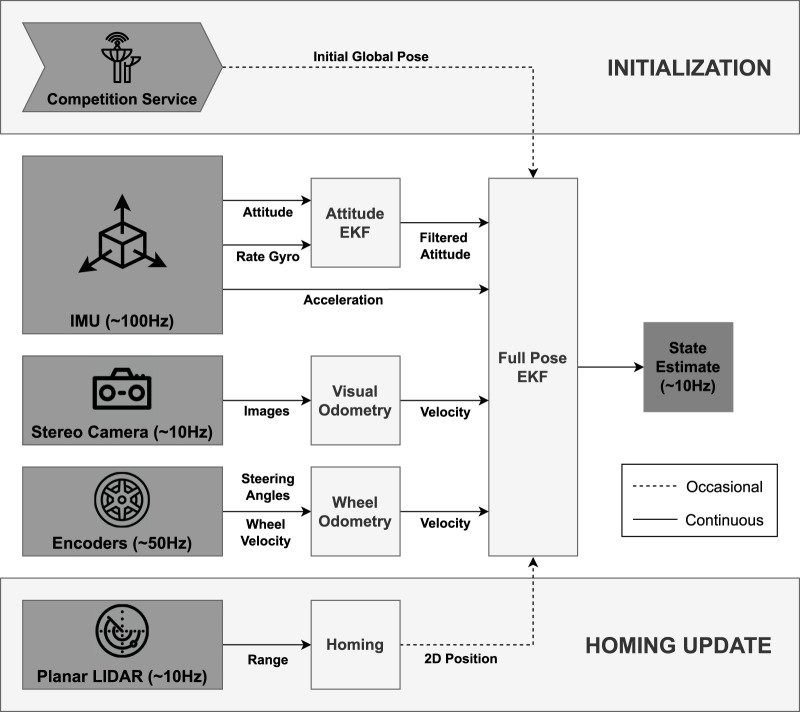
The architecture of the state estimation framework as is in our previous work (Kilic et al., 2021). Initialization of the state of each rover was performed at the beginning of a competition run. A homing update was performed periodically to improve localization estimation. A standalone EKF was dedicated to providing a filtered attitude estimation. Velocity information from the wheel and stereo camera is used in a sensor fusion EKF to provide the rover velocity and position estimations. Finally, rover full state estimation (attitude, velocity, position) was generated by the combination of the data coming from these two different sources.

Our localization and navigation architecture for the competition round was based on the successful Qualification Round implementation, as detailed in our previous work (Kilic et al., 2021). The main differences between the Qualification and Competition rounds that affected the previous architecture were the requirement for accurate localization with noisy sensors within two hours of operation, whereas the simulation runtime for the Qualification Round was 45 min, and the need for rover recharging in the competition. The increased operation time led to position error accumulation that needed to be addressed by the rovers to perform reliable Lunar *In-Situ* Resource Utilization (ISRU). Additionally, for recharging, the rover solar panels needed to be oriented correctly with respect to the Sun, or the rovers needed to be near the recharging station. For completeness, the architecture of the implemented state estimation framework is depicted in Figure 3 and the critical points of this implementation are summarized as:

1. State estimation was performed utilizing an extended Kalman Filter (EKF) (Ribeiro, 2004) that consists of fusing wheel odometry (WO), visual odometry (VO), and a standalone attitude estimation EKF.
2. The localization performance was improved with periodic homing updates with the recharging station. The recharging station had a cylindrical shape, and its center was registered in the global frame as a global landmark. For this, the 2D LiDAR data was used in a least-squares estimator to fit the data to a circle, assuming the rover has accurate global attitude estimation.
3. Autonomous navigation tasks of the rovers were done making use of Move Base framework for multi-robots (Pütz et al., 2018).
4. A waypoint navigation strategy is used to explore the virtual map, searching for the randomly distributed volatiles.

Because of the difficult terrain conditions presented in the initial simulation, the decision was made not to utilize SLAM throughout the competition. When SLAM packages were utilized, because the terrain was featureless and dark, we observed an unsatisfactory loop closure performance. As a result, a technique based on homing updates was established to achieve loop closure instead. More features were added to the terrain as the competition progressed, such as a more realistic texture and lighting, which allowed other teams to effectively deploy SLAM packages. However, by that stage, our solution had already met the localization objectives and our attention had shifted to other competition challenges.

### 4.2 Driving control

The three types of rovers shared the same motion system hardware, four-wheel steering (4WS), with passive suspension. The steering angle and wheel speed could be controlled independently. The maximum wheel speed was 10 rad s^−1^ and the steering angles ranged from −180^◦^ to 180^◦^. In our approach, we implemented a controller that selected one from three different locomotion modes using body velocity commands accordingly. The three locomotion types were pure translation, pure rotation, and a combination of translation and rotation.

A body velocity command consists of three fields: linear speed on the longitudinal axis (*v*_*x*_), linear speed on the lateral axis (*v*_*y*_), and rotational speed on the vertical axis (*ω*_*z*_). Pure translation, also known as synchronous-drive or crab motion (Li et al., 2022), was selected by the driving controller when *ω*_*z*_ = 0. In this mode, all the wheels are steered to an angle that produces the desired combination of *v*_*x*_ and *v*_*y*_. Pure rotation, also known as point turns or turn-in-place maneuver was selected by the driving controller when only *ω*_*z*_ > 0. In this mode, all the wheels are steered to have their axles aligned with the radii of circles of a common center point located at the vehicle’s center of gravity.

A Double Ackermann steering (Qiu et al., 2018) system was used to combine translation and rotation. This mode was selected by the driving controller when *v*_*y*_ = 0 and *v*_*x*_ > 0 and *ω*_*z*_ > 0. In this mode, all the wheels are steered to an angle that produces the desired combination of *v*_*x*_ and *v*_*y*_. In this mode, all the wheels are steered to have their axles aligned with the radii of circles of a common center point located at a distance from the vehicle’s center of gravity (in the center of rotation point).

This multi-modal locomotion approach was chosen to optimize the motion for different parts of the challenge. For example, a simple skid-steering approach can provide pure rotation and the combination simultaneously; however, the resulting wheel slip degrades the localization estimation, especially when trying to turn in place (Zuccaro et al., 2017). The different locomotion modes are shown in Supplementary Figure S1.

Finally, the simulation also provided a braking service, with the option of braking from 0% to 100%. At 100%, a braking torque of 500 N m rad^−1^ was delivered to each wheel simultaneously. A linear ramp with adjustable time intervals was used to stop the rovers. This adjustable time to go from 0% to 100% braking was fine-tuned during the tests to stop the rover without causing excessive wheel slippage.

### 4.3 Mobility hazard detection and immobility recovery

The main aim of the rover mobility hazard detection and immobility recovery system was to keep the rover safe and fully operational to maximize the volatile exploration area coverage and excavation. Even though the environment was mostly traversable, there were potential physical threats to the rovers randomly distributed in the environment, such as craters, small hills, and rocks. The navigation subsystem could avoid these threats; however, occluded small obstacles near craters and small hills on the map, marked as traversable in some cases, endangered the rover operations with possible tipping over and becoming stuck cases. Considering these threats, several precautions to keep the rovers safe were included for each type of rover. For example, the Scouts were programmed to drive as safely as possible by limiting their traversability over craters and rocks, assuming their speed could compensate for the extra waiting time for path planning. Further, the Scouts had no explicit requirement to reach the goals given by the waypoints because their main goal was to explore the area to find the volatiles.

Haulers and Excavators were programmed to follow strict navigational goals since they needed to pinpoint the excavation area for a successful digging operation. During testing, the rovers successfully fulfilled their operational needs. However, sporadic failures on any other system could lead to a cascade effect and compromise the rover’s mobility. For example, whenever the visual odometry node cannot generate a disparity image, it could interfere with the obstacle detection node and lead to the inclusion of artifacts in the cost map used by the navigation node, resulting in the rover assuming that it was stuck on the terrain. In addition, when the navigation stack was disengaged for any blind driving maneuver (e.g., during an approach maneuver), the navigation stack could be resumed at a location that the rover believes to be coincident with an obstacle, which makes the navigation stack unable to provide a navigation plan.

To overcome these topological and algorithmic threats, we developed a rover mobility hazard detection and immobility recovery system that could sense if any further rover operation was unsafe or if the rover was unable to move due to being stuck. When such a situation is detected, the rover performs several predetermined recovery maneuvers.

The immobility sensing was performed in a localization watchdog system where the wheel slip was calculated and classified into one of five known cases. The reason for having five known cases in the immobility sensing approach is to provide a multi-layer of safety that properly identifies the immobility state of the rover. In these cases, we leveraged the ratios between commanded velocities *versus* estimated velocities, commanded steering angles *versus* actuated wheel steering angles, and wheel odometry estimates *versus* visual odometry estimates. Detecting the wheel slippage or steering angle anomaly was performed by an iterative indicator that accumulated slip and wheel anomalies triggering the rover to declare immobility after a certain threshold. After the declaration, the rover would perform recovery maneuvers to try to recover. In addition to immobility, the state machine incorporated a pitch and roll indicator that halts the rover and drives backward to generate a new plan if the estimated pitch or roll exceeds a predetermined safety threshold. This threshold was determined to be 27^◦^ based on the Scout’s center of gravity during the tests and helped prevent the rover from flipping or rolling over due to excessive pitch or roll.

**FIGURE 4:**
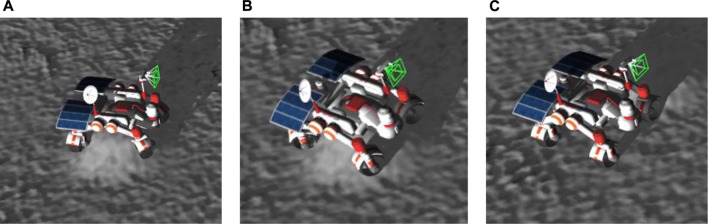
Stuck detection capability. **(A)** Stuck detection. **(B)** Recovery maneuver. **(C)** Mobility recovered.

In a case of being stuck, which is a case when the rover tried to traverse over a small hill, and all its wheels lost contact with the terrain, whereas the rover frame was placed on top of the hill, the rover started performing unstuck recovery maneuvers. The main idea of recovering the stuck rover was to make the rover wheels regain contact with the terrain. For this, we leveraged the geometric specifications of the wheels, given that the diameter of the wheels is longer than the width of the wheels, and utilized the four-wheel steering capability of the rover. The maneuver was performed by steering the wheels to gain traction at any point on the hill’s surface enabling the rover to move and become mobile again. An example scenario of the recovery after getting stuck is given in Figure 4.

The Mobility Hazard Detection and Immobility Recovery system was developed to assure the rovers’ safety and their full operating capabilities in a challenging environment with possible physical dangers such as craters, steep slopes, and rocks. The system identified any unexpected rover activity or immobility and executed specified recovery operations, such as regaining traction and becoming mobile again by exploiting the geometric specifications of the wheels and the rover’s four-wheel steering capacity. The system allowed the rovers to function safely and effectively, thus meeting their operational requirements.

### 4.4 Manipulation

In order to reduce computational complexity and to perform excavating actions within the mission time limit, a simple approach where the kinematic equations were used to help control the motion of the arm from predefined configurations to the target points consisting of the volatile position or Hauler’s bin was used. First, coordinate frames were assigned to each joint, and then Denavit-Hartenberg parameters were obtained. With this information, forward and inverse kinematics relations were derived for the arm.

The forward kinematics formulation is straightforward for this 4R manipulator and the equations can be obtained geometrically from the link lengths and joint angles. The inverse kinematics was also obtained geometrically by using two orthogonal, uncoupled planes of motion: one considers changing the azimuth of the whole arm (shoulder yaw), and the other considers changing the configuration of the arm (shoulder pitch, elbow pitch, wrist pitch) in the z-r plane. The desired shoulder yaw joint angle was obtained directly using the cylindrical coordinates and the other joint angles were obtained using a standard geometrical method used for 3R planar manipulators. More details on this approach can be found in Kilic et al. (2021).

After solving the Excavator arm’s forward and inverse kinematics there are several ways to plan its motion. The planning constraints included: 1) avoiding collisions and 2) maintaining the bucket’s global angle within a specific range to ensure that the volatile clods were collected from the terrain and not dropped unintentionally. Predefined configurations were selected to act as safe waypoints for the arm to guarantee that there will be no collision during the motion. Between them, trajectories were obtained by interpolating starting and ending joint angles using third-order polynomial trajectories.

To know if the desired position (a point on top of the Hauler’s bin) was reachable, we derived the workspace of the arm through range and height maps given all possible shoulder pitch and elbow pitch angles. A depiction of these maps can be seen in Supplementary Figure S2. This considers that any yaw angle was reachable and the wrist joint would be actuated to maintain the bucket aligned with the horizon. These maps were interpolated and functions were included for quick access inside the Manipulation stack. Specific scenarios, for example, requiring shoulder yaw angles that would lead to a collision with the Excavator’s camera, were dealt with separately, and range and height were further restricted for them.

### 4.5 Object detection

The goal of the object detection system is to help perceive the environment surrounding the robot, allowing them to interact with it autonomously. The most critical functions include detecting relevant objects and features in the camera images and also estimating geometric information with respect to the camera frame. In our approach, the Single Shot Multi-Box Detector (SSD) (Liu et al., 2016) is used to detect, in real-time, bounding boxes around target objects appearing in the robot camera images. The algorithm was chosen due to its detection performance and real-time inference capabilities. When using SSD, the inference can be processed at 60 Hz. The detection was implemented using a centralized approach.

A custom algorithm was developed to select, synchronize, and process images for object detection. Specifically, this algorithm synchronizes stereo pairs and selects images from each robot. Later, it feeds them to a single SSD neural network. In sequence, it organizes the data output from the multi-box detector, distributing back the information that is relevant to each robot, such as the bounding box locations or the filtered point cloud of an object. Using this approach, all six rovers were able to obtain information about the environment in real-time because each of the robot camera images was obtained at 5 Hz from the simulator.

**FIGURE 5:**
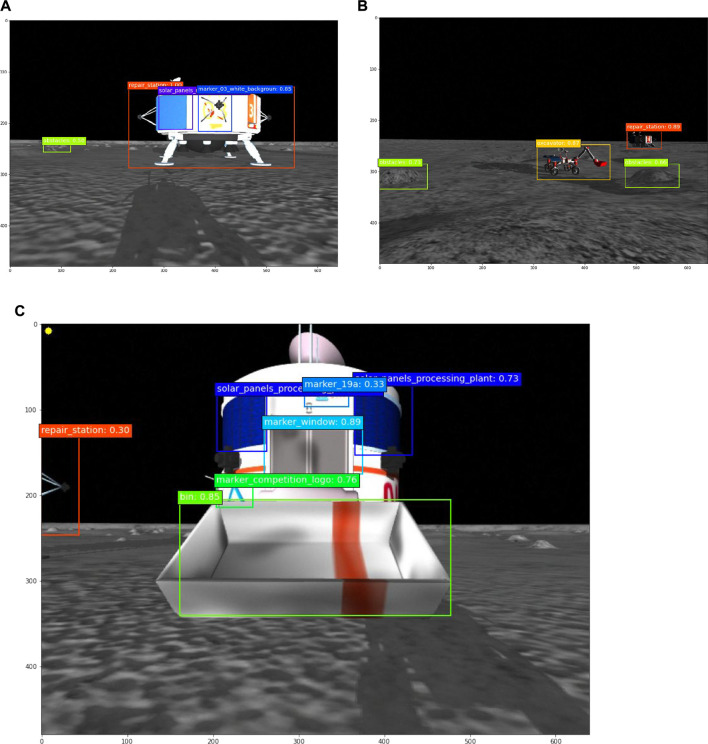
Object detected during approach sequence. **(A)** Charging station. **(B)** Excavator and obstacles. **(C)** Processing plant and its bin.

An SSD 300 network, based on the VGG16 architecture, was chosen due to its compatibility with the size of the dataset obtained for training. This network takes 300 × 300 × 3 normalized RGB images as inputs. The bounding box regression is similar to Szegedy et al. (2014). A set of pre-defined bounding boxes, called feature maps are connected to different layers from the VGG16 network so that the different scales of feature maps are processed at different layers. The network estimates the bounding box position and its class in a single shot. Therefore, the loss function is divided into two parts. The classification loss, which is the object class, is minimized using categorical cross-entropy, and the location loss, which is how far the predicted bounding boxes are from the ground truth is minimized using smooth L1-Norm Girshick (2015). The output bounding boxes are pruned using non-maximum suppression, where only 400 boxes with a confidence of more than 0.01 and intersection over the union of more than 0.7 are kept. The network was pre-trained using the Microsoft Common Objects in Context (MS COCO) (Lin et al., 2014), which consists of 350,000 images and 80 object categories. Afterward, weights were sampled for 22 new classes of objects seen in the competition environment. These classes include the three types of rovers, two types of obstacles, the processing plant, the charging station, and specific features of these objects, such as the competition logo, solar panels, and the Excavator’s bucket. The dataset was collected from images randomly sampled from camera image recordings of the robots being teleoperated in the simulation environment for more than 6 h. A total of 5,629 images were manually annotated and used as training data. Another 1,119 images were annotated and used as validation. The image data reproduce the situations that the rovers would encounter when deployed to perform their tasks autonomously, such as approaching other rovers and landmarks and driving through obstacles. Because the diversity of objects in the lunar environment is small and restricted, and every object is known, overfitting was not a major concern during training. Figure 5 shows examples of bounding box detection with images captured by the camera.

The disparity images from the stereo camera pair were calculated using semi-global block matching (Hirschmuller, 2005). Then, using the bounding box coordinates and stereo camera parameters, each pixel inside the object bounding box was used to calculate the 3D point cloud. Because artificial noise was added to the simulated images, outlier points were removed. Also, points with a *z* coordinate (optical axis) values larger than a threshold of 20 m were discarded due to the low quality of images (640 × 480), which caused estimation to perform poorly above that distance.

The processed data was pipelined to the navigation system of the rovers in two ways. The obstacles’ point clouds were sent continuously to the MoveBase node of each rover, guaranteeing safe driving. The bounding boxes for objects of interest and the corresponding estimated point clouds were sent on demand to the ROS node that provided the control during approach maneuvers for each rover when they had to interact with each other and the stations.

### 4.6 Approach procedures

Besides obstacle avoidance, visual perception was mainly used for target-approaching maneuvers. The approach procedures started with the rover at a waypoint near its goal followed by controllers based on vision and LiDAR to approach reliably. Three types of approaches were performed during the mission:

(1) A Hauler approaching an Excavator for volatile collection,
(2) A Hauler approaching a processing plant to dump the volatile and score points,
(3) Any rover approaching the charging station for recharging and updating rover localization with the LiDAR.

The three types of approaches followed similar steps. The first step was to rotate in place to identify the object we aimed to approach. Once the object’s bounding box is found, the rover moved forward, centralizing the box in the center of the image. LiDAR and disparity images were used to estimate the distance from the object and to decide when to stop. Given the features of the data, some objects were better identified with lights on and some with lights off. So, the rover lights were toggled on and off to maximize the chances of identifying features. Additional maneuvers were developed to handle multiple robot interactions and coordination in order to increase success rates. For example, to coordinate the behavior of two rovers when they both needed to access the base stations, a flag that was triggered when the base station was needed. This would act as a stoplight to prevent a second rover from approaching at the same time. Further, to handle a rover crossing in front during a visual servoing approach, a stop was triggered anytime another robot appeared in front during the approach drive. Another coordination that was developed was to have a rover drive backward 0.5 m for the robot to better align the rover with the goal and provide sufficient space to maneuver. Figure 5A shows the bounding boxes seen by the Scout during an approach to the charging station, Figure 5B shows the bounding boxes seen by a Hauler during the maneuver to approach an Excavator, and Figure 5C shows the bounding boxes seen by the Hauler during the approach bin maneuver.

## 5 Autonomous operation and coordination

**FIGURE 6:**
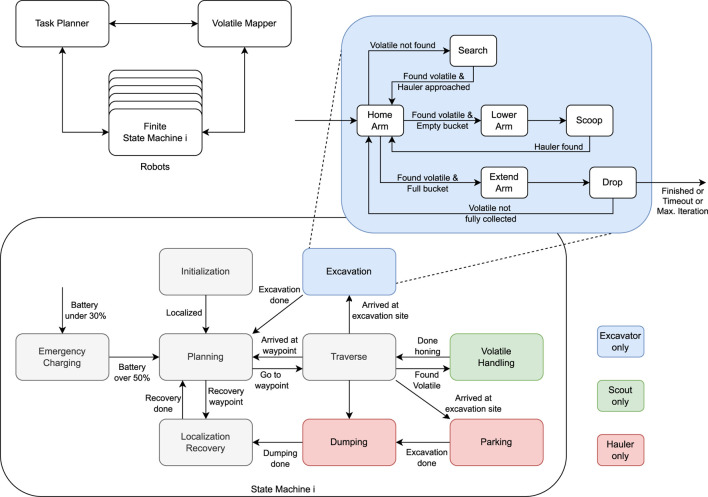
Coordination architecture for autonomous operation. A central task planner communicates and has access to the states of each individual robot, and resource collection states from a shared volatile map. The goals and resource states are updated by the task planner and the robots as their operations progress. Each robot has its behavior controlled by a Finite State Machine. Three kinds of finite state machines (FSMs) were developed, one for each type of rover. Most of the states are common (gray) to the three kinds of FSM, however, each type of rover had specialized states (colored) related to their tasks. For the Excavator, another FSM was nested to control the manipulator behavior.

This section provides detailed explanations of the developed approaches and their specific usage cases with demonstrations to overcome the competition challenges. Additionally, this section describes how each approach presented in Section 4 works in harmony to facilitate the multi-robot system to perform the lunar ISRU mission in the SRCP2 challenge. The framework for the autonomous operation in our solution consisted of a central task planner and volatile map, and decentralized finite state machines (FSMs) to control individual robots. The task planner was responsible for allocating waypoints for the scouts, excavation sites for excavators and haulers, and coordinating the access to dumping at the processing plant. The volatile map was tasked with recording the location and status of the resources found on the map. All the rovers had their operation controlled by individual FSMs, as depicted in Figure 6.

Most of the FSM states were common to the three types of rovers, namely, “Initialization”, “Planning”, “Traverse”, “Localization Recovery” and “Emergency Charging” states. During the “Initialization” state, the rovers initialized their localization estimates, as described in Subsection 4.1, and were spread out in the map to predefined locations to reduce the chances of rover-to-rover and rover-to-infrastructure interactions at the beginning of their operation. Next, they proceed to the “Planning” state and waited for a goal. Upon receiving a goal, they proceeded to the “Traverse” state, which traversed them to the location that a task planner desired them to be. This location can be, for example, an exploration waypoint for a Scout, an excavation site for an Excavator, or a waypoint to aid in dumping for a Hauler. After reaching their goal, they can either switch back to the “Planning” state and wait for another goal or proceed to a specialized task such as excavating. The “Emergency Charging” state was activated immediately if the rover battery dropped under 30%. The “Localization Recovery” state was activated for Scouts upon receiving a recovery waypoint and for Haulers after each dumping. This state was used for resetting the localization estimates using a homing strategy and recharging the batteries at the charging station.

The remaining FSM states were specific to each type of robot, depending on their mission. The “Volatile Handling” state was present only for Scouts, the “Excavation” state was present only for Excavators, and the “Parking” and “Dumping” states were present only for Haulers. The “Volatile Handling” state was activated whenever a Scout detected volatile material in the map and tried to minimize the distance of the robot to the location of the resource. When initiated, the “Excavation” state activated another FSM that was for controlling the actions of an Excavator’s arm to search, excavate, and drop volatile clods in the Hauler’s bin. The Haulers used their “Parking” state to approach and park close to their partner Excavator and the “Dumping” state to haul the excavated material back to the processing plant. The following sections provide more details regarding the task planner developed and the operation of the three types of robots.

### 5.1 Central task planning and volatile mapper

Mission task planning consisted of assigning waypoints to each robot when requested. This was centralized in a Task Planner ROS node. The two Scouts performed a preprogrammed search of the field with regular stops for homing. To reduce the immobility risks on harsh terrain, Scouts could skip waypoints except for homing. The two Excavators were sent to the next closest available volatile. At the start of the mission, special handling was developed to ensure that the two excavations were performed within a reasonable distance. Therefore, if the first two volatiles were found close to one another, the second excavation team would wait for the third volatile to be discovered. For communication simplicity, each Hauler was partnered with a specific Excavator. Their waypoints from the task planner were assigned to be the same as their partner Excavator, slightly offset by a distance (10 m). The volatile mapper ROS node was tasked with recording the volatile resources that were found by the Scouts and keeping track of their properties and status. The properties of the volatile resources included the index, type, position, and minimum distance measured to the corresponding Scout. The status of the volatile resource was recorded in regards to whether the resource had been previously observed if an attempt had been made to collect it, and if the collection was successful or unsuccessful.

### 5.2 Scout mission and autonomy

The scouts were equipped with a dedicated sensor that is able to detect volatiles below the Lunar surface. This sensor is able to detect any volatiles within a 2 m radius. It returns (as a ROS message) a noisy measurement of the distance to the center of the closest volatile, and its type (i.e., ice, ethane, methane, methanol, carbon dioxide, ammonia, hydrogen sulfite, or sulfur dioxide). Since the dedicated volatile sensor could only detect the volatiles at such a short range with respect to the rover, keeping a reliable and continuous localization solution for the rover played a critical role in correctly reporting the resource locations and reaching the desired waypoints for exploration. Dedicated FSMs (Figure 6) were used to control each of the Scouts. In the “Initialization” state, the Scouts’ localization filters were initialized using the initial positions provided by the competition. The global heading angle was the same for all six robots and was obtained using one of the available position fixes (i.e., a single-use competition-provided true pose service). For each Scout, the provided position fix was used during the detection of the second volatile, which pinpointed the location of the second excavation site. The rover state transitioned to “Planning” upon completion of the initialization phase.

#### 5.2.1 Exploration strategy

Approximately one hundred waypoints were used for exploring the inner region (|*x*| < 50, |*y*| < 50) of the map. We decided to focus on this region because it was observed that the volatile distribution was denser closer to the center of the map. The map was divided into two-halves and each Scout was tasked with exploring one of them. This strategy minimized possible interactions and overlaps in their exploration area. Exploration routes were designed to start and end close to the center of the map and a sequence of waypoints was distributed along the routes. By finishing an exploration route at the center of the map, the robot would be in proximity to the recharging station plant such that a homing update could be performed before the robot enters the next exploration plan.

In the “Planning” state, the robot requested a waypoint from the task planner node. If there was no collision in its field of view, the waypoint was passed to the navigation framework, and the state was transitioned to traverse. In the “Traverse” state, the rover drives from one waypoint to another using navigation and driving subsystems (see Subsections 4.1, 4.2). The “Localization Recovery” state aimed to minimize the possible failures in planning and traversing states. If the rover was experiencing immobility issues, the recovery state was triggered by the stuck detection and steep slope detection. Also, when the navigation plan was not achievable in the “Planning” state, the rover reset its current plan and switched to the “Planning” state to generate a new navigational plan.

#### 5.2.2 Volatile honing strategy

When a volatile was sensed during driving by a Scout, the “Volatile Handling” state was activated. During this state, the rover reported the location of the volatile using its localization solution while considering the lever arm of the mounting location of the volatile sensor with respect to the center of localization (e.g., volatile sensor location to IMU location). For volatile reporting, the rover uses the logic to anticipate the volatile position by a honing strategy moving sideways and forward to minimize the distance between the rover and the volatile until the rover naturally finds its minimum distance to the volatile.

**FIGURE 7:**
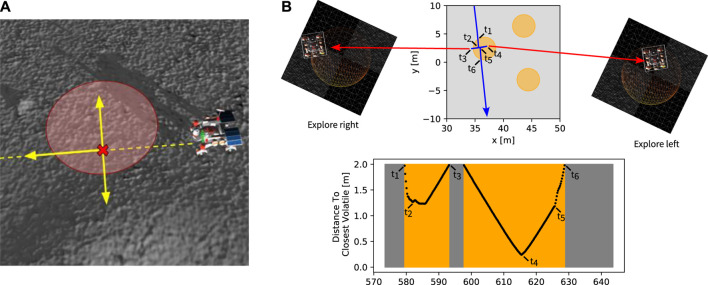
Honing Procedure. **(A)** Along its exploration trajectories (yellow dashed line), a Scout can find a volatile and will perform maneuvers to guess the position of the center of the resource. **(B)** The scout will decrease its speed and continue on its original trajectory while the sensed distance decreases (between timestamps *t*_1_ and *t*_2_). Once the distance increases (*t*_2_), it expects the volatile to be to its left or right, orthogonally to the exploration trajectory. In sequence, it will move a few meters to the left (*t*_3_) and to the right (*t*_4_) to hone the reported volatile position. Once the sideways maneuvers end (*t*_5_), the Scout resumes its exploration trajectory with and regular speed (*t*_6_).

The honing strategy is demonstrated in Figure 7. During the traverse, a Scout follows a path represented by the dashed yellow line (Figure 7A). Once a volatile is detected, the Scout has its speed sharply reduced. In our approach, the Scout stops at a position marked with the cross in (Figure 7A) once it naturally gets to the minimum distance to the center of the volatile while still following its original path. Then it drives sideways to both sides before going back to moving forward Ideally, this strategy would achieve near zero minimum detected distance. In reality, when the slip and rough terrain are involved, we observed that the Scouts were able to achieve a minimum detected distance at the decimeter level (Figure 7B) while not altering the Scout’s trajectory or odometry solution significantly.

#### 5.2.3 Homing update

After visiting some predetermined locations, each Scout performs homing by activating the “Localization Recovery” state. In this state, the rover drives to the charging station, approaches it with visual servoing, and performs a localization update. The localization update used LiDAR to fit a circle to the cylindrically shaped charging station and estimate the location of the circle’s center with respect to the robot’s position. Given that the charging station is a static landmark (i.e., its location is known), it was possible to use the difference between the estimated position and the true position to get a correction vector. Additionally, with this information we were able to reject the result when the estimated radius was smaller or larger than the expected radius of the recharging station. Likewise, because we used a least squares estimator to determine the fit to a circle, the magnitude of the residuals of the fit was used to eliminate outliers. In particular, if a rock or some other object were present and obstructed the circle fit, the estimation residuals were very large and the estimate could be rejected. In these instances, we simply did not use the update and waited until the next recharging. These checks were empirically tuned. This LiDAR-based approach was only used when the station was present in the camera image.

In the absence of a loop-closure strategy such as, for example, periodic homing update, the localization error of the robotic system is likely to significantly increase due to the challenging terrain characteristics (i.e., steep slopes and low features), leading to wheel slippage and visual odometry (VO) failures (Gonzalez et al., 2018; Strader et al., 2020; Kilic et al., 2022). Consequently, any localization inaccuracy issue can yield consecutive unsuccessful reports for the sensed volatiles. Even though some of the homing updates could be unnecessary for localization error mitigation; these updates provided considerable assistance in keeping the rover’s localization accuracy sufficient to map the sensed volatiles.

### 5.3 Excavator mission and autonomy

The principles behind the Excavator mission consisted in going to a position determined by the task planner where a Scout has previously found a volatile. Dedicated FSMs (Figure 6) were used to control each of the Excavators. As excavation sites become available, an Excavator was sent a goal through the “Planning” state and navigates to that location using the “Traverse” state. Once the Excavator arrives at the excavation site, it activates the “Excavation” state. First, during this state, it defines an appropriate parking position for the Hauler by analyzing its surroundings. Afterward, it initiates the digging process, actively searching for the found volatile. Since there are accumulated localization errors (self and mapped volatile location), it needs to search in a region using some driving maneuvers before digging. Upon successfully locating the volatile resources, the Excavator communicates with the Hauler that it may proceed with its approach maneuver. Then, once the Hauler indicates that the approach is complete, the Excavator utilizes its camera to scan the area and employs object detection services to identify and determine the position of the Hauler’s bin. The excavation process continues if the bin position is reachable, and volatile can be dropped into the bin. If not, the Excavator asks its Hauler for a re-parking maneuver. After successfully identifying that volatile can be deposited at the Hauler’s bin, the Excavator executes a series of arm trajectories based on the relative heading and range of the target bin. At each scoop, the Excavator reconfirms the Hauler’s bin position to guarantee that the relative localization is still valid since skidding may cause drifts. The digging process ends once one of the following conditions applies: the volatile is fully captured, the volatile clods cannot be found anymore, a maximum number of scoops is reached (set to 12), or the entire process reaches a timeout (about 20 min in simulation time). The most important parts of this procedure are detailed next.

#### 5.3.1 Deciding the best side to park

Once the Excavator reaches (or thinks it reaches) the position of a volatile, it rotates its camera 360 degrees and, by counting the number of obstacles identified on each of its sides (left or right), it decides the best side for the Hauler to park by counting the number of large rock objects are detected on each side of the excavation side. It then computes a pose perpendicular to the Excavator, 10 m away at the chosen side, and communicates it to the Hauler, which uses that pose as a set point. The Hauler will wait in this position until the Excavator indicates it needs to approach.

#### 5.3.2 Searching for volatile

After finding the best side to park the Hauler, the Excavator inserts the scoop into the ground and checks if a volatile is present. If a volatile is found, the Excavator moves to the next step. If a volatile is not found, the Excavator removes the scoop from the ground and starts a search procedure within the maximum detection radius of 1.5 m around the current point. It moves in eight directions to cover the region to detect the volatile specified by the angles 0, 45, −45, −90, 90, 135, −135, and 180 degrees. For each position, the Excavator searches for the presence of volatile by inserting and removing the scoop in the ground. The Hauler is commanded to approach if the volatile is found. If the volatile is not found after this procedure, the excavation is canceled, and the team waits for a new volatile location to be sent by the Task Planner (Subsection 5.1).

#### 5.3.3 Finding the partner Hauler

After successfully finding the volatile, the partner Hauler is commanded to park on one of the sides of the Excavator. However, the Excavator does not know the exact position the Hauler is parked, given the meter-level inaccuracies of the position estimate of the robots. To mitigate this problem, the Excavator rotates its camera until it finds the Hauler. The Hauler is detected in the camera image based on the color and the size of the Hauler bin, which is easily distinguished from other objects around the robot. Supplementary Figure S3 shows the Hauler as seen by the Excavator’s camera. Finally, the region containing the bin is masked on the depth image obtained from the stereo pair. It is used to compute the relative 3D position of the bin with respect to the Excavator with centimeter-level accuracy. This position is used in the next excavation step.

#### 5.3.4 Excavation state machine

**FIGURE 8:**
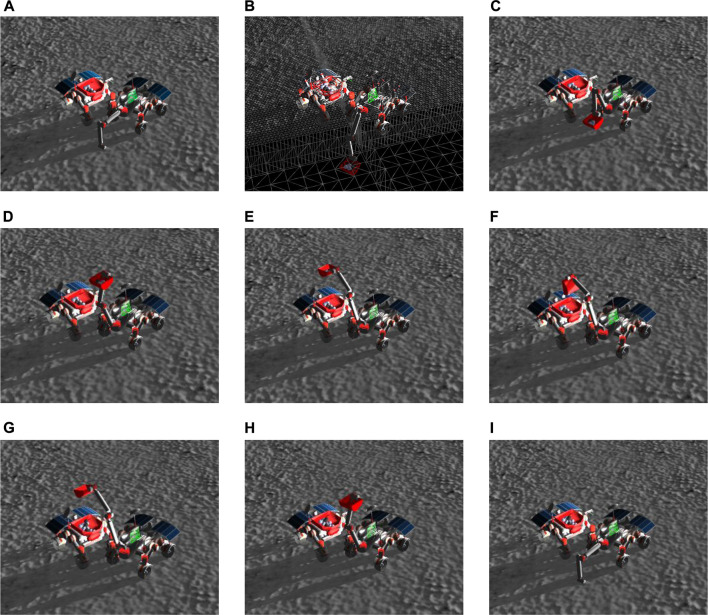
Excavation procedure is achieved by a sequence of actions followed by the Excavator’s arm. **(A)** The Excavator lowers its arm and identifies the position of the Hauler’s bin. **(B)** The arm is lowered to scoop for volatile clods, which are spawned 0.4 m below the terrain level. **(C)** The arm is taken to a Home position to prevent collisions with the Hauler’s wheels. **(D)** The arm is raised to avoid collisions with the Excavator’s camera when moving to a specific heading. **(E)** The arm is extended on top of the Hauler’s bin. The range and heading of the bin define the joint angles. **(F)** The bucket is emptied, and the volatile is dropped into the Hauler’s bin. **(G)** The arm is retracted to a safe position. **(H)** The arm is brought back to the front of the Excavator. **(I)** The process starts again. It loops until no more volatile is found.

Once the Excavator reached the location close to the volatile that needed to be excavated, it enabled a secondary state machine to actuate the arm, as shown in the top right of Figure 6. Its states, namely, “Home Arm”, “Search”, “Lower Arm”, “Scoop”, “Extend Arm”, and “Drop”, had predefined actions associated with them to simplify the manipulation motion planning problem. The “Home” state is used as an intermediate waypoint to many different trajectories. The “Search” state executes a search pattern (see Subsection 5.3.2), tells the Hauler that it has found volatile, and then starts a waiting period for the Hauler to park at the Excavator’s side. Once the Hauler parks, the Excavator transitions to the “Scoop” state. During the “Scoop” state, the first step was to lower the arm below the terrain and try to find the Hauler’s bin with the “FindHauler” service (see Subsection 5.3.3). Once it finds the Hauler, it scoops material and checks if volatile is detected in the Excavator’s bucket. If there is, it proceeds to an “Extend” state that extends the arm in the direction of the Hauler’s bin, and finally, to a “Drop” state that turns the bucket and drops the contents. This sequence is repeated until all the mass of the volatile is collected. The sequence of actions is exemplified in Figure 8.

#### 5.3.5 Communication with Hauler

Two custom ROS messages were created to coordinate the actions between a Hauler and an Excavator team during an excavation process. These messages were used to transmit tasks status and target waypoints between the excavators and haulers. The excavation process begins when the Excavator identifies a suitable parking spot on its left or right side and sends this information to the Hauler along with a target waypoint for the Hauler to arrive at. The Hauler then informs the Excavator by sending a message that indicates the Hauler is approaching the Excavator. Once the Hauler has reached the target side waypoint, it relays a message to the Excavator indicating that it has approached the target site. After this, the Hauler waits for the Excavator to report that the volatile was found and ready to excavate. The waiting process for the Excavator message is necessary since the Excavator may need to search the local area to find the volatile.

Once the Excavator reports that it has found the volatile, the Hauler starts its parking sequence to reach the identified parking spot through a visual approach (approached Excavator flag), then gets an estimate of the global Excavator location in its reference frame using either computer vision (primary) or laser (secondary) and communicates with the Excavator indicating that it is attempting final parking. Once the Hauler ensures that it has successfully parked (parked Hauler flag) the Excavator uses its camera to find the Hauler’s bin, as discussed in the Subsection 5.3.3. Once the bin has been identified and determined to be in a suitable location, the excavator proceeds with excavation, utilizing a number of scooping actions. In the event that the Hauler bin is not detected or is determined to be too distant, a parking recovery protocol is initiated. This process includes requesting a parking recovery and setting a “failed to find Hauler” flag. The excavation process then restarts, beginning with the approach phase, up to a maximum of three attempts as determined by the parking recovery counter. If the Excavator is unable to locate volatile, the Excavator-Hauler team requests a new excavation site from the task planner, and the Hauler returns to the charging station to recharge its battery and re-establish its localization.

### 5.4 Hauler mission and autonomy

**FIGURE 9:**
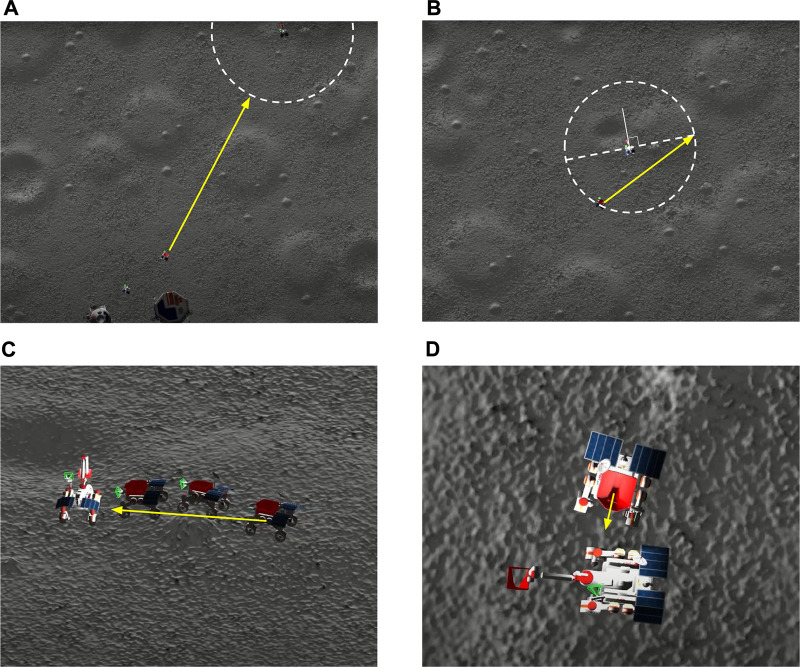
Parking Procedure. **(A)** Once the Excavator arrives at the excavation site, the Hauler drives using its navigation stack and stops at a radius (white dashed circle) from the volatile position in a staging waypoint. Then it waits for new instructions from the Excavator about the best side to park. **(B)** The Excavator chooses which side it wants the Hauler to park and the Hauler goes to this waypoint, again using its navigation stack. It stops at a 10 m range and perpendicularly to the Excavator’s orientation. Again it waits for the Excavator to instruct it to start approaching. **(C)** After finding volatile the Excavator requests the Hauler to approach. The Hauler approaches using visual servoing until it gets to a 3 m range. **(D)** Then it tries several methods to determine a precise position to park. After deciding the best position to park, the Hauler goes to that position using a position-feedback velocity controller and it waits until the load is fully deposited in its bin by the Excavator.

The Hauler’s mission was designed to work in conjunction with its partner Excavator at an excavation site. During the “Planning” state, it receives a designated staging area from the Task Planner (see Subsection 5.1). It switches to the “Traverse” state and moves until that goal is reached. Next, it waits until the Excavator indicates from which side the Hauler should approach (as described in Subsection 5.3.1. The Hauler then moves to a waypoint located 10 m to the left or right of the Excavator. This distance is predetermined by tests to give the Hauler a safe maneuvering space before reaching the parking spot. Next, the Hauler activates its “Parking” state and proceeds with parking at the side that was selected by the Excavator. These procedures are illustrated in Figure 9. Once the excavation process ends, the Hauler switches to the “Dumping” state, is commanded to leave the site and begins transporting the extracted volatile clods back to the processing plant. The Hauler proceeds to an intermediate waypoint located in front of the processing plant. To avoid interference between two haulers wanting to dump its bin at the same time, we implemented a simple “semaphore”. This way, the Hauler requests permission from the Task Planner to proceed with unloading the materials, which will only be granted if no other Haulers are currently unloading. Once permission is granted, the Hauler executes a sequence of actions that result in the unloading of the volatile clods into the processing plant’s bin. The Hauler then proceeds to a charging station where it corrects its localization using a homing update and recharges its batteries. Finally, the Hauler traverses to the next excavation site to repeat the process.

#### 5.4.1 Parking procedure

Once the Hauler arrives at the side waypoint that the Excavator chose, it rotates in place in order to align its camera with the Excavator. When the Excavator finds a volatile, it commands the Hauler to start approaching. This procedure (explained in Subsection 4.6) detects the Excavator in Hauler’s camera image and drives towards it using visual servoing. This approach stops the Hauler at a 3 m range from the Excavator, a distance that guarantees that the Excavator showed up with sufficient detail in the depth image and LiDAR measurements. In sequence, the Hauler tries to park very close to the Excavator, so that the Excavator’s arm can reach the hauler bin. Then, three methods are attempted sequentially. First, the Hauler tries to estimate a parking position using computer vision. The Excavator raises its bucket, the Hauler checks for a red blob of the right size in its camera image, and from there a parking location is calculated. If this method fails, the Hauler tilts its sensor suite up and down, to generate a 3D scan of the Excavator with its LiDAR. Then, it estimates the Excavator position and, from this, a parking location. If these two methods fail, the robot falls back to using a visual servoing approach to park near the Excavator. These methods are illustrated in Supplementary Figure S4. Once the Hauler believes it has parked, based on the relative position feedback, it sends a message to the Excavator. The Excavator verifies that the Hauler is parked by reciprocally estimating the relative position of the Hauler bin. If the Hauler bin is detected and its position is estimated to be within a specified range (as described in Subsection 5.3.3), the Excavator has confirmed that the Hauler is parked and excavation can be continued.

### 5.4.2 Dumping procedure

**FIGURE 10:**
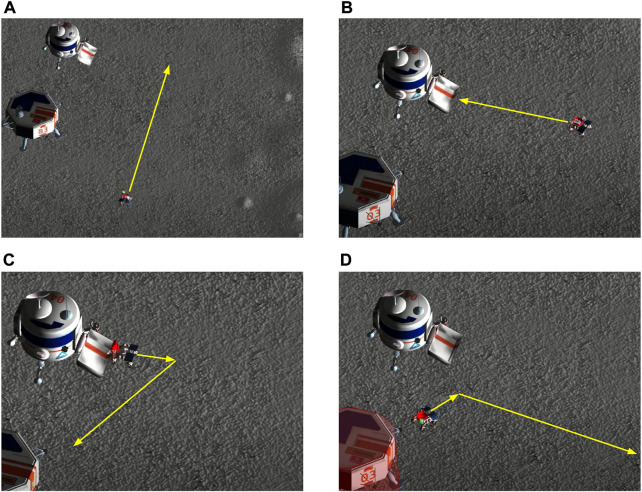
Dumping Procedure. **(A)** Once the excavation is complete, the Hauler drives to a predetermined staging waypoint using its full navigation stack including obstacle avoidance. **(B)** After arriving at the staging waypoint, visual servoing approach is used to guide the robot to the processing bin. The Hauler has a slight forward velocity when against the bin to ensure proper alignment and executes a deposit. **(C)** After depositing the materials a blind backup maneuver is executed. During this entire sequence of events, the other Hauler would not be allowed to approach the processing bin by coordinating through the task planner. The Hauler then enters a new state to approach the charging station to refill batteries. Another visual approach is used to align the robot with the charging station. **(D)** Once at the charging station, a LiDAR-based homing update is performed to reduce localization errors. After this step, the Hauler proceeds to the following excavation site. During this period, its partner Excavator has already been traversing to this next site, finding a suitable parking spot for Hauler, and confirming the presence of volatile material.

The dumping procedure starts when the Excavator communicates that the excavation is over to the Hauler. Then, the Hauler backs up a couple of meters to give a safe space to the Hauler for performing maneuvers and drives to a waypoint in front of the processing plant. When it arrives at this waypoint, if permission to proceed is granted by the Task planner, it calls the service to approach the processing plant’s bin using visual servoing to drive the rover closer (*ApproachBin* service). Once it is there, it activates its bin and dumps the contents. Due to localization errors, sometimes it is possible that the known waypoint in front of the processing plant is not reached. This would lead to a poor approach. To mitigate this, we included another service to find the bin using the camera images, and estimate the relative location of the processing plant’s bin (*FindBin* service). Since the robot orientation is measured directly and noise is reduced using the Attitude EKF (see Subsection 4.1), it is possible to know if the robots are outside of the region where the *ApproachBin* service would work well. When this happens, *FindBin* also generates another intermediate waypoint whose coordinates (in the odometry frame of the rover) will take the rover to the front of the processing plant. From that waypoint, the *ApproachBin* service is executed normally. After dumping the contents, the Hauler proceeds to the charging station to refill its batteries and execute Homing, to correct its localization estimates. This procedure is illustrated in Figure 10.

## 6 Results and discussion

To evaluate the overall performance of our system, we first show the results of a single run in more detail, and then we share the results obtained from our actual submission to the challenge evaluation system. Each run consisted of 2 h of simulation time, however, using our computer setup (desktop with an Intel i9-9900K Octa-core (8 Core) 3.60 GHz Processor, 32 GB RAM, NVIDIA GeForce RTX 2080), the simulation took around 10 h in real time to be complete.

### 6.1 Two-hours sample run

During each simulation run, six robots were operated in a randomly generated terrain world. On a typical run, all tasks described before were performed successfully and consistently. However, many unexpected events occurred due to uncertainties, unanticipated logical sequences, interactions, or behaviors. For example, some of the excavation processes were not completed successfully for a variety of reasons including incorrectly mapped volatiles, poor Excavator localization, difficulties with parking, failed scoop dumps, robot collisions, and harsh terrain.

**FIGURE 11:**
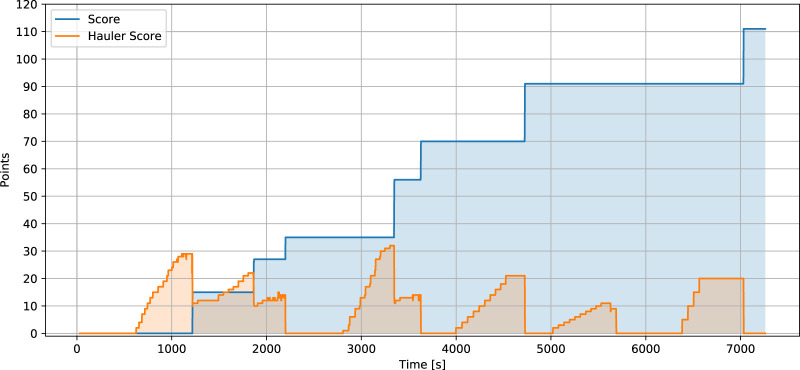
Score achieved during a two-hours mission with six robots.

Figure 11 shows the points collected over time for this run. When points are collected by a Hauler, a Scout was able to detect a volatile and report its position on the map. Subsequently, an Excavator and a Hauler moved to that site, and the Excavator searched, dug, and dumped the volatile material in the Hauler’s bin. When points are scored, the Haulers were able to transport the collected resources back to the processing plant. In this particular run, our solution was able to complete this maneuver seven times, which resulted in a score of 111 points.

**FIGURE 12:**
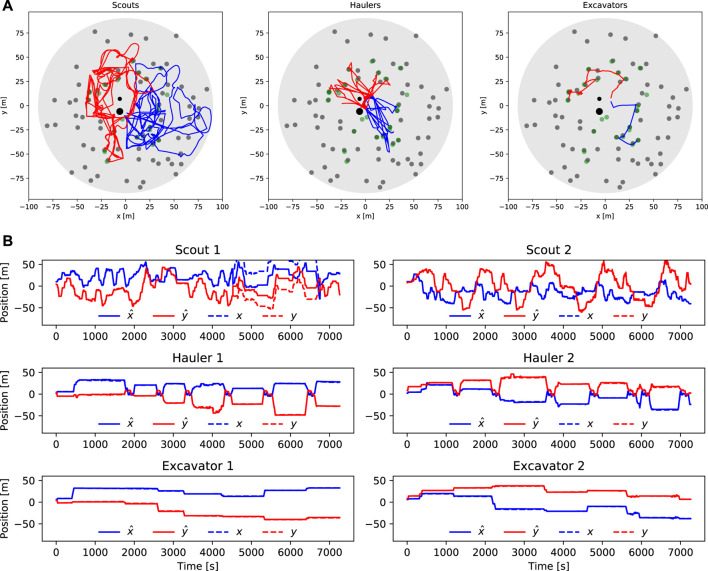
Localization during a two-hours mission for multiple robots. **(A)** Truth trajectories during the two-hours simulation for all six robots. **(B)** Estimated (solid lines) and truth (dashed lines) trajectories during the two-hours simulation for all six robots.

Figure 12 shows the trajectories of the six rovers during a simulation run. Figure 12A shows the location of the volatiles in the map and the trajectories that the different kinds of robots executed. It also shows the reported volatiles in green. It is possible to see that the Scouts’ trajectories cover the area on the map and that the failure observed on the localization estimate for Scout 1 led to a set of wrongly reported volatiles. It also shows how the Excavators were sent to a sequence of volatiles given by proximity and Haulers were also sent to these places and back to the processing plant to deposit the resources.

The x and y coordinates of the rovers are shown in Figure 12B. The Scouts’ graphs show more jagged trajectories since they are moving all around their sides of the map. Scout 1 searches the positive-x half side of the map, while Scout 2 searches the negative-x half side until they shift sides between timestamps 4,550 and 6,750. The Haulers and Excavators have matching positions. However, the Haulers, every so often, move to the coordinates of the processing plant to dump the clods in their bin. Excavators barely move. Most of the time they are still, waiting for a new goal or digging. We observed that the small drifts in localization error were caused by the movement of the rovers in slippery terrain and the integration of noisy measurements in the localization filter. In general, the big jumps in localization errors were caused by one of the following: collision with other robots or landers, getting stuck in rocks, and incorrect homing updates. While Scout 2 did not experience any of these problems during this simulation, it was subject to small drifts, which were corrected with good homing updates. Scout 1, on the other hand, experienced one of these big jumps. Our procedure for correction of the localization was based on homing, and that was performed every 4 or 5 waypoints given to the navigation stack. In this simulation, Scout 1 had to go through these waypoints before calling the homing update service that used visual servoing to drive to the base station. Notice that the visual servoing approach is able to work even without a localization solution because it only relies on the camera images to find or drive to the station. Once the Scout got to the base station, it was able to correct its localization estimate to meter level and go back to functioning correctly. We chose this simulation run specifically to exemplify some of the features that were added to the robots’ behaviors to provide robustness in our solution.

**FIGURE 13:**
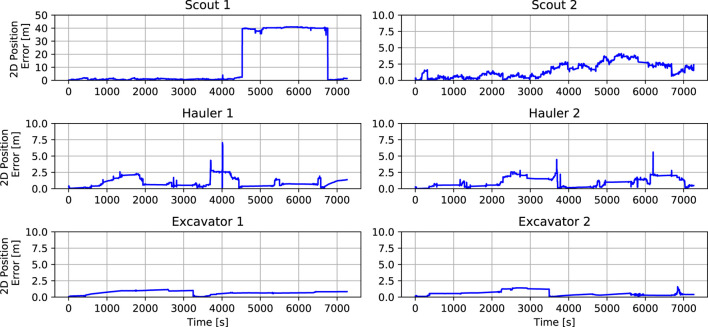
Horizontal error during a two-hours mission for multiple robots.

Figure 13 shows the localization error for the six rovers. The visual inertial odometry (VIO) solution slowly drifts from the ground truth when the rovers move. The Excavators barely move during the run, so they do not need to return to the repair station to recharge their batteries and perform homing. They are able to recharge using solar panels only and maintain a reasonable localization estimate. The Haulers and Scouts move more often, and their localization estimates are kept bounded ( < 5 m) by doing frequent homing, allowing for much longer operation time. Scout 1 shows an interesting case where an error occurs and it completely loses its localization estimate. However, by doing homing, it is able to re-localize itself and become functional again.

**FIGURE 14:**
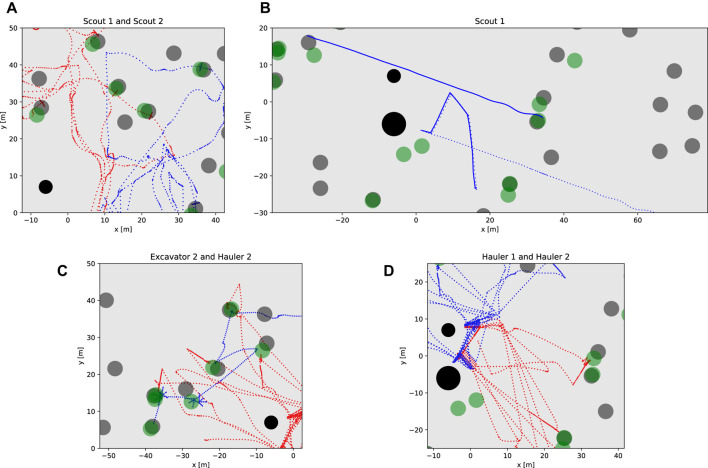
Rover operations in detail. The volatiles true position is shown in dark grey and the position recorded by the scouts is shown in green. **(A)** Volatile search and mapping for Scout 1 (blue) and Scout 2 (red). **(B)** True (dashed) and estimated (solid) trajectory for Scout 1 when it performs a homing update to correct its localization estimate. **(C)** Excavator (blue) and Hauler (red) visiting sites to excavate and transport resources. It is possible to see that the excavator had to use its 8-directions search at least twice to find volatiles. **(D)** Hauler dumping and fast recharge.

Figure 14 shows a portion of the Scouts, Excavators, and Haulers’ truth trajectories to illustrate some of their behavior. Figure 14A shows the two Scouts executing search maneuvers whenever volatile was detected, trying to minimize the distance measured by the volatile sensor to the location of its center. Figure 14B shows the localization estimate (solid line) and the actual trajectory executed by Scout 1 from 6,500 s to 6,900 s. At 6,725 s, Scout 1 updates its localization estimate using the homing update at the repair station and the horizontal position error dropped to 0.3 m. Notice on Figure 14C that the Excavator (blue dots) sequentially visited sites on the map that were reported by a Scout. The first three sites were well-reported, and the Excavator was able to find the resources in its first dig. On the following two sites, it performed an 8-directional grid search. For the first one, it was not able to find the resource and it gave up, proceeding to the next, where it was able to find the resource. It is also possible to notice that the Hauler (red dots) parked at a distance and slowly approach the Excavator as described in Sect. 5.4.1. In Figure 14D it is possible to see the Haulers’ movements to stop in front of the processing plant, approach the processing plant bin, back up, move to the charging station, and move to the following excavation site.

**FIGURE 15:**
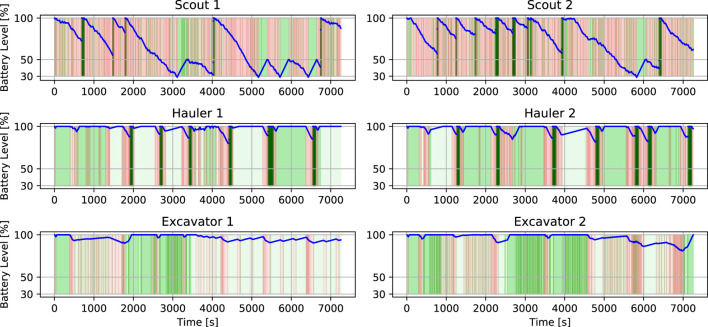
Battery management during a two-hours mission for multiple robots.

Figure 15 shows the battery level for all the rovers during the run. The background color indicates the rate of change in the battery level. During most of the operation, the rovers charge and discharge in the interval −1 (red) to 1 W (green), depending on its action and solar panels status. Whenever any of the rovers approach the repair station, the battery rate becomes 200 W (dark green), and the battery level increases almost instantaneously to the maximum level. This figure shows the difference in battery management for the different types of robots. Given that the Scouts’ task requires constant traverse, the repair station was used both for improving localization and charging them. The top graphs also show that whenever the Scouts’ batteries drop to 30%, they go to a charging state and recharge to 50% before proceeding. By doing so they can eventually reach the repair station and fully recharge. The Haulers do not move as much as Scouts, since they are waiting to be filled most of the time. Their battery level only changed when they traverse the map to drop when their bins’ contents in the processing plant and headed back to the excavation site. However, we took advantage of the fact that the repair station was close to the processing plant and had the Haulers fully recharged before going back to the excavation site. The Excavators only move between excavation sites, and very infrequently. Their battery level was maintained nearly full by slowly charging with the solar panels.

Table 1 shows the main metrics for this sample run. The ROS bag is provided in our Supplemental Data. In this particular run, our solution was able to score about three times the minimum requirement. The Scouts were able to map more than 24 volatiles in each round. The Excavator and Hauler teams were able to attempt the collection of around 14 volatiles, however, they were only successful in collecting 8 out of the 14 resources attempted.

**TABLE 1:** Sample run metrics.

Score	Hauler score	Number of resources types collected at conclusion	Robots working at conclusion	Volatiles		
				Mapped	Attempted	Collected
111	0	4 (CO_2_, H_2_O, H_2_S, C_2_H_6_)	6	25	14	8

### 6.2 Competition performance

The methodology was submitted for testing in July 2021. The competition returned back the results from an independent analysis of our solution. The solution was tested in three different randomized maps. As shown in the competition rule document (NASA Centennial Challenges Program (CCP), 2021), the main criterion for deciding the winners of the challenge was scoring the largest number of points at the end of the simulation. A minimum required score of 35 points was stipulated by NASA for the solution to be considered. The tie-breaking criteria ordered by importance, were 1) the least combined mass of the virtual robotic team, 2) the point value of resources in haulers, 3) the number of resource types collected, and 4) the number of original robotic team members still performing their intended function(s).

Table 2 shows the main metrics demonstrating the success of our solution. The ROS bags and logs are provided in our Supplemental Data. Our solution was able to score about two times the minimum requirement, with an average score of 69.6 points. According to the logs, immobility recovery was triggered for two robots, and was able to recover their functionality. However, in one of the rounds, a Scout became ceased working properly by the end of the simulation. The Scouts were able to map more than 15 volatiles in each round. The Excavator and Hauler teams were able to attempt the collection of around 15 volatiles, however, they were only successful in collecting 8 out of the 15 resources attempted.

**TABLE 2:** Final submission metrics.

	Score	Hauler score	Number of resources types collected at conclusion	Robots working at conclusion	Volatiles		
					Mapped	Attempted	Collected
Map 1	63	12	2 (H_2_O, SO_2_)	6	15	14	8
Map 2	83	0	4 (C_2_H_4_, H_2_S, H_2_O, CH_3_OH)	6	23	15	8
Map 3	63	0	4 (H_2_S, H_2_O, CH_4_, SO_2_)	5	17	16	8

## 7 Conclusion

This paper introduces the robotic systems and the autonomous operation methodology used by our team to solve the NASA Space Robotics Challenge Phase 2. We explained the difficulties that can be encountered in a lunar environment and how they can be overcome with our solution, which successfully demonstrated the operation and cooperation of six robots autonomously for two hours. In this complex multi-robot mission, we presented possibilities to navigate Scouts, Excavators, and Haulers; improve their global and relative localization, and their localization estimation recovery; detect and correct mobility failures.

While our solution was able to generate good results, it still had several limitations. The best score we obtained was 217^1^. Arguably, this is around the maximum score our solution could achieve: during that simulation, the excavators were able to find all the resources that were assigned to them, and haulers were able to transport them back to the processing plant safely. However, we are aware that the team ranked second scored up to 403 points (Brabec, 2021), and the team ranked third scored up to 339 points (Sachdeva et al., 2022). Despite implementing an effective localization system for this environment, we opted not to utilize SLAM with loop closures, cooperative localization techniques between rovers, and slip compensation techniques, which may have the potential further improve our localization solution. On another note, the mobility of the rovers was substantially impacted by the high level of slippage resulting from challenging obstacles in the environment, which required the rovers to perform a homing update in order to regain their localization. To minimize the immobility risks, the rovers were driven slower than their achievable speeds which resulted in having less exploration time on the map for the scouts.

During the competition, several key lessons were identified. One such lesson was the realization that utilizing simpler strategies may lead to increased scoring. For example, since the scouts were driven slowly, the strategy was to maximize their exploration capabilities by moving them as frequently as possible without stopping them at a location. In this strategy, once a scout located a volatile, the position information was recorded and indexed, and then a state machine was used to determine which excavator-hauler team should be dispatched to the location. Rather than relying on localization estimates from the scouts, an alternative approach could have been to use the scouts as landmarks for the excavators after a volatile location detection. This would have allowed for more precise volatile location identification and reduced the time required for searching in the reported location, while also mitigating the impact of localization drift on excavating operations. Another lesson learned was regarding the use of FSMs for many robots and states. FSMs lack modularity and reactivity (Colledanchise and Ögren, 2018). Their complexity can grow very quickly and software development can quickly get concentrated on a few people that can understand them. Communication between FSMs is also complex and, in our experience, resorts to a large number of Boolean flags, opening up for unaccounted logical sequences and leading to failures. Alternative approaches to FSMs, like the use of behavior trees, were successfully implemented by another winning team (Burtz et al., 2020).

To improve our solution some future work could include 1) the implementation of a multi-objective optimization to decide which type of resources found would be excavated first and by which team, 2) the implementation of a terrain assessment or terrain semantic segmentation to improve obstacle detection and avoidance, 3) implementation of visual SLAM algorithm to provide another layer of robustness to our localization system, 4) employment of reinforcement learning techniques for excavating and dumping the resources, and 5) implementation of behavior trees to reduce the complexity of controlling the behavior of individual rovers and their interaction.

## Data Availability

The datasets and software presented in this study can be found in online repositories. The names of the repository/repositories and accession number(s) can be found below: https://drive.google.com/drive/folders/1iMt95EzX6Twft95_fr4c5XCgkIS6-pnU?usp=sharing.

## Footnotes

1
https://www.youtube.com/watch?v=NhOFn3zNXbA

## References

[B1] Asnani V. Delap D. Creager C. (2009). The development of wheels for the lunar roving vehicle. J. Terramechanics 46, 89–103. 10.1016/j.jterra.2009.02.005

[B2] Brabec F. (2021). Space robotics challenge phase 2 electronic summary team robotika. Available at https://bit.ly/3GX7SN7 (Accessed 01 2023, 20).

[B3] Burgard W. Moors M. Stachniss C. Schneider F. E. (2005). Coordinated multi-robot exploration. IEEE Trans. robotics 21, 376–386. 10.1109/tro.2004.839232

[B4] Burtz L. Dubois F. Guy N. (2020). “Human-robot teaming strategy for fast teleoperation of a lunar resource exploration rover,” in International symposium on artificial intelligence, robotics and automation in space (Lunar and Planetary Institute).

[B5] Chien S. Barrett A. Estlin T. Rabideau G. (2000). “A comparison of coordinated planning methods for cooperating rovers,” in Proceedings of the fourth international conference on Autonomous agents, 100–101.

[B6] Colaprete A. Andrews D. Bluethmann W. Elphic R. C. Bussey B. Trimble J. (2019). “An overview of the volatiles investigating polar exploration rover (viper) mission,” in AGU fall meeting abstracts, 2019, P34B–P03.

[B7] Colaprete A. Elphic R. Andrews D. Trimble J. Bluethmann B. Quinn J. (2017). “Resource prospector: An update on the lunar volatiles prospecting and ISRU demonstration mission,” in Proceedings of the 48th lunar and planetary science conference (Woodlands, TX, USA: LPSC), 20–24.

[B8] Colledanchise M. Ögren P. (2018). Behavior trees in robotics and AI: An introduction. CRC Press.

[B9] Fong T. Bualat M. Deans M. Allan M. Bouyssounouse X. Broxton M. (2008). “Field testing of utility robots for lunar surface operations,” in AIAA SPACE 2008 conference and exposition, 7886.

[B10] Fong T. (2021). “Nasa autonomous systems and robotics: Roadmap and investments,” in Lunar surface innovation consortium fall 2021 meeting.

[B11] Girshick R. (2015). “Fast r-cnn,” in Proceedings of the IEEE international conference on computer vision, 1440–1448.

[B12] Gonzalez R. Apostolopoulos D. Iagnemma K. (2018). Slippage and immobilization detection for planetary exploration rovers via machine learning and proprioceptive sensing. J. Field Robotics 35, 231–247. 10.1002/rob.21736

[B13] Hirschmuller H. (2005). “Accurate and efficient stereo processing by semi-global matching and mutual information,” in 2005 IEEE computer society conference on computer vision and pattern recognition (CVPR’05) (IEEE), 2, 807–814.

[B14] Khamis A. Hussein A. Elmogy A. (2015). Multi-robot task allocation: A review of the state-of-the-art. Coop. robots Sens. Netw., 31–51.

[B15] Kilic C. Gu Y. Gross J. N. (2022). Proprioceptive slip detection for planetary rovers in perceptually degraded extraterrestrial environments. Field Robot. 2, 1754–1778. 10.55417/fr.2022054

[B16] Kilic C. Martinez B. Tatsch C. A. Beard J. Strader J. Das S. (2021). NASA space robotics challenge 2 qualification round: An approach to autonomous lunar rover operations. IEEE Aerosp. Electron. Syst. Mag. 36, 24–41. 10.1109/maes.2021.3115897

[B17] Li W. Yang F. Mao E. Shao M. Sui H. Du Y. (2022). Design and verification of crab steering system for high clearance self-propelled sprayer. Agriculture 12, 1893. 10.3390/agriculture12111893

[B18] Lin T.-Y. Maire M. Belongie S. Hays J. Perona P. Ramanan D. (2014). “Microsoft COCO: Common objects in context,” in European conference on computer vision (Springer), 740–755.

[B19] Liu W. Anguelov D. Erhan D. Szegedy C. Reed S. Fu C.-Y. (2016). “Ssd: Single shot multibox detector,” in European conference on computer vision (Springer), 21–37.

[B20] Mahdoui N. Frémont V. Natalizio E. (2018). “Cooperative frontier-based exploration strategy for multi-robot system,” in 2018 13th annual conference on system of systems engineering (SoSE) (IEEE), 203–210.

[B21] NASA (2020a). 2020 NASA technology Taxonomy. Available at https://www.nasa.gov/offices/oct/taxonomy (Accessed 02 2023, 20).

[B22] NASA Centennial Challenges Program (2021). Space robotics challenge – phase 2 (SRC2) official rules. Available at https://bit.ly/3wj1T0p (Accessed 09 2022, 20).

[B23] NASA Space Technology Mission Directorate (2020). Lunar surface innovation initiative. Available at https://go.nasa.gov/3D2zBej (Accessed 09 2022, 20).

[B24] NASA (2020b). The Artemis plan, NASA’s lunar exploration program overview. Available at https://go.nasa.gov/3WrOLjW (Accessed 09 2022, 20).

[B25] NASA’s Marshall Space Flight Center (2021). Teams develop code to coordinate robots, win $535,000 in space robotics challenge. Available at https://go.nasa.gov/3GX8hiB (Accessed 01 2023, 20).

[B26] Nishida S.-I. Wakabayashi S. (2012). Lunar surface exploration using mobile robots. Open Eng. 2, 156–163. 10.2478/s13531-011-0072-z

[B27] Open Robotics (2022). ROS documentation. Available at http://wiki.ros.org/(Accessed 09 2022, 20).

[B28] Polizzi V. Hewitt R. Hidalgo-Carrió J. Delaune J. Scaramuzza D. (2022). Data-efficient collaborative decentralized thermal-inertial odometry. IEEE Robotics Automation Lett. 7, 10681–10688. 10.1109/lra.2022.3194675

[B29] Portugal D. Rocha R. P. (2013). Distributed multi-robot patrol: A scalable and fault-tolerant framework. Robotics Aut. Syst. 61, 1572–1587. 10.1016/j.robot.2013.06.011

[B30] Putz P. (1998). Space robotics in Europe: A survey. Robotics Aut. Syst. 23, 3–16. 10.1016/s0921-8890(97)00053-5

[B31] Pütz S. Simón J. S. Hertzberg J. (2018). “Move base flex,” in 2018 IEEE/RSJ international conference on intelligent robots and systems (IROS) (IEEE), 3416–3421.

[B32] Qiu Q. Fan Z. Meng Z. Zhang Q. Cong Y. Li B. (2018). Extended ackerman steering principle for the coordinated movement control of a four wheel drive agricultural mobile robot. Comput. Electron. Agric. 152, 40–50. 10.1016/j.compag.2018.06.036

[B33] Ribeiro M. I. (2004). Kalman and extended kalman filters: Concept, derivation and properties. Institute for Systems and Robotics 43, 46.

[B34] Sachdeva R. Hammond R. Bockman J. Arthur A. Smart B. Craggs D. (2022). Autonomy and perception for space mining. In 2022 international conference on robotics and automation (ICRA) (IEEE), 4087–4093.

[B35] Schenker P. S. Huntsberger T. L. Pirjanian P. Baumgartner E. T. Tunstel E. (2003). Planetary rover developments supporting Mars exploration, sample return and future human-robotic colonization. Aut. Robots 14, 103–126. 10.1023/a:1022271301244

[B36] Schenker P. S. Huntsberger T. L. Pirjanian P. Trebi-Ollennu A. Das H. Joshi S. S. (2000). “Robot work crews for planetary outposts: Close cooperation and coordination of multiple mobile robots,” in Sensor fusion and decentralized control in robotic systems III (SPIE), 4196, 210–220.

[B37] Skasiadek J. (2013). Space robotics and its challenges. Aerosp. Robot., 1–8.

[B38] Smith M. Craig D. Herrmann N. Mahoney E. Krezel J. McIntyre N. (2020). The Artemis program: An overview of NASA’s activities to return humans to the Moon. In 2020 IEEE aerospace conference, 1–10. 10.1109/AERO47225.2020.9172323

[B39] Stachniss C. Stachniss C. (2009). Coordinated multi-robot exploration. Robotic Mapp. Explor., 43–71.

[B40] Staudinger E. Shutin D. Manß C. Viseras A. Zhang S. (2018). “Swarm technologies for future space exploration missions,” in ISAIRAS’18: Fourteenth international symposium on artificial intelligence (ROBOTICS AND AUTOMATION IN SPACE).

[B41] Strader J. Otsu K. Agha-mohammadi A.-a. (2020). Perception-aware autonomous mast motion planning for planetary exploration rovers. J. Field Robotics 37, 812–829. 10.1002/rob.21925

[B42] Szegedy C. Reed S. Erhan D. Anguelov D. Ioffe S. (2014). Scalable, high-quality object detection. *arXiv preprint arXiv:1412.1441*

[B43] Team Capricorn (2021). GitHub Repository WPI-NASA-SRC-P2/capricorn_competition_round. Available at https://bit.ly/3GT3z5v (Accessed 01 2023, 20).

[B44] Team L3 (2022). GitHub repository TeamL3/learned-pose-estimation/. Available at https://bit.ly/3ZKdYt3 (Accessed 01 2023, 20).

[B45] Thangavelautham J. Xu Y. (2022). “The design of autonomous robotic technologies for lunar launch and landing pad (llp) preparation,” in 2022 IEEE aerospace conference (AERO), 1–13.IEEE

[B46] Weisbin C. R. Rodriguez G. (2000). Nasa robotics research for planetary surface exploration. IEEE Robotics Automation Mag. 7, 25–34. 10.1109/100.894030

[B47] Zakrajsek J. McKissock D. Woytach J. Zakrajsek J. Oswald F. McEntire K. (2005). “Exploration rover concepts and development challenges,” in 1st space exploration conference (Continuing the Voyage of Discovery), 2525.

[B48] Zuccaro S. G. Canfield S. L. Hill T. W. (2017). “Slip prediction of skid-steer mobile robots in manufacturing environments,” in International design engineering technical conferences and computers and information in engineering conference (American Society of Mechanical Engineers (ASME)), 58172, V05AT08A036.

