# Supplementary material for: Multi-robot cooperation for lunar In-Situ resource utilization
Source: Front Robot AI. 2023 Mar 23;10:1149080. doi: 10.3389/frobt.2023.1149080 (PMC10076576; doi:10.3389/frobt.2023.1149080)
Supplement: Supplementary file 1 [file DataSheet1.pdf]

frontiers

# *Supplementary Material*

## 1 SUPPLEMENTARY TABLES AND FIGURES

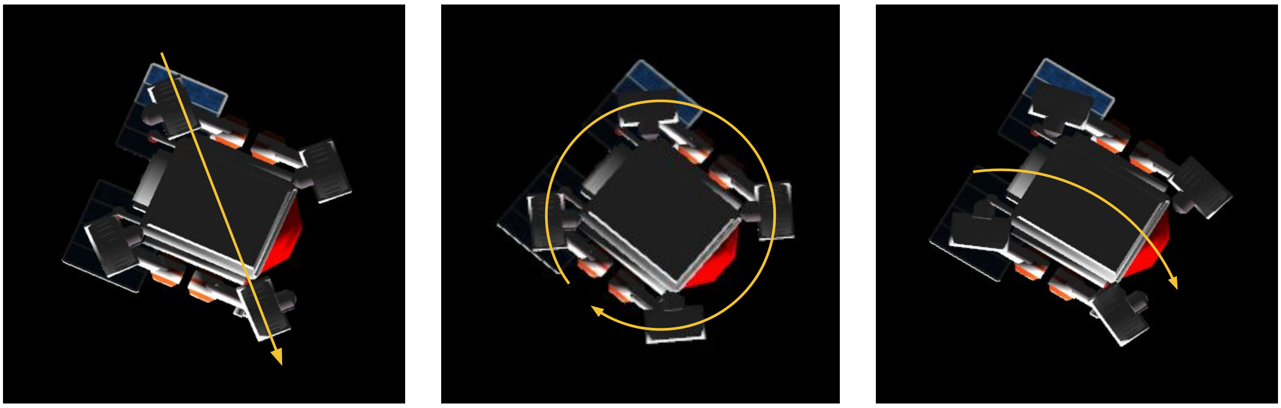

**Figure S1.** Locomotion Modes. **(A)** Pure translation mode. **(B)** Pure rotation mode. **C** Double Ackermann steering.

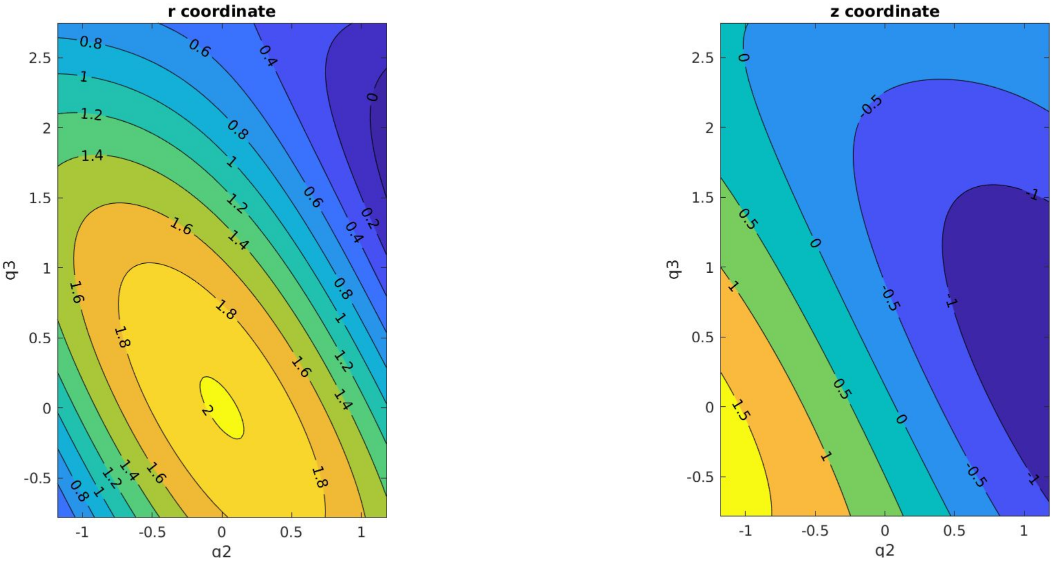


**Figure S2.** Workspace of the Excavator's arm was parameterized by the shoulder ($q_2$) and elbow pitch ($q_3$) joints. This allowed for quick checking if a desired goal for the arm is reachable and if the Hauler needs to park again. **(A)** Radial range map ($r$ coordinate). **(B)** Height map ($z$ coordinate).

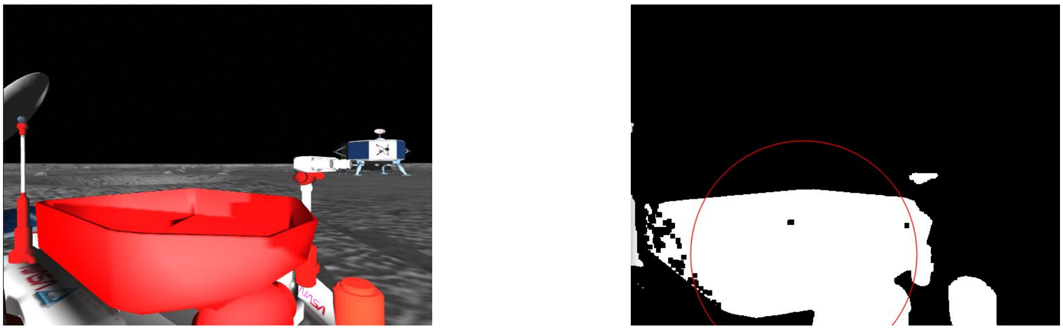

**Figure S3.** Detection of the Hauler's bin. The Excavator finds the Hauler bin by processing the original RGB image using color segmentation. **(A)** RGB Image captured by the Excavator. **(B)** Detector mask.

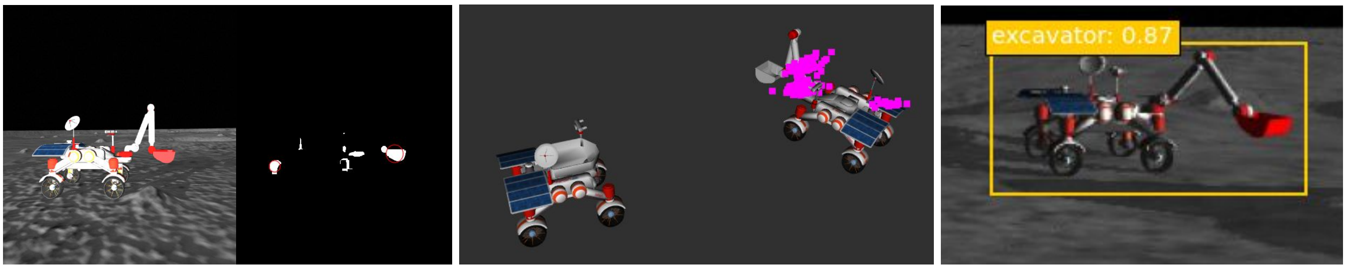


**Figure S4.** Methods for estimation of the relative 3D position between Hauler and Excavator. **(A)** Using blob detection and depth image segmentation. **(B)** Using an assemble of filtered laser scans. **(C)** Using a real-time object detection system and depth image segmentation.

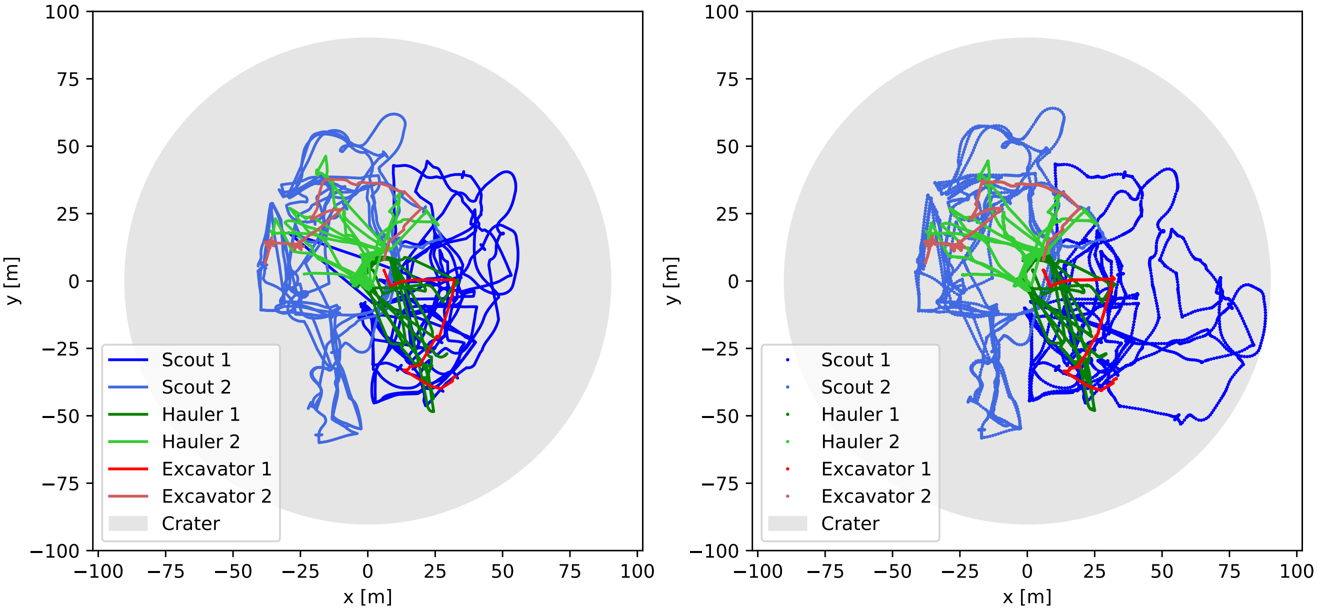


**Figure S5.** Comparison between **(A)** our localization estimate and **(B)** the localization truth for a two-hours mission for multiple robots.
